# Exploring 130 years of temperature-related mortality in the city of Madrid

**DOI:** 10.1038/s41598-026-38595-4

**Published:** 2026-02-25

**Authors:** Dariya Ordanovich, Diego Ramiro, Aurelio Tobias

**Affiliations:** 1 Institute of Economy, Geography and Demography, Spanish National Research Council, Madrid, Spain; 2 Institute of Environmental Assessment and Water Research, Spanish National Research Council, Barcelona, Spain

**Keywords:** Risk factors, Climate-change adaptation, Climate-change impacts, Environmental health

## Abstract

The relationship between ambient temperatures and health outcomes has been extensively studied, yet long-term analyses of mortality responses to extreme temperatures, particularly in the Mediterranean region, remain scarce. This study leverages daily all-cause mortality and air temperature data in the city of Madrid from 1890 to 2019 to examine adaptation patterns to extreme and moderate heat and cold. Using a distributed lag non-linear modeling framework, we explored temperature-mortality relationships and estimated decade-specific adaptation metrics. Results indicate a general reduction in temperature-attributable mortality, primarily due to decreased cold-related risks across all population groups. While moderate heat impact declined over time, extreme heat effects remained stable, with a slight increase towards the study’s end. Our findings highlight the dual impact of climate change, i.e. reducing cold-related mortality while stabilizing heat-related risks, which emphasizes the complex interplay of climate change and health outcomes and offers new insights into adaptation dynamics using long-term temperature-mortality time series.

**Supplementary Information:**

The online version contains supplementary material available at 10.1038/s41598-026-38595-4.

## Introduction

The temperature-mortality association has been extensively studied in the past decades, especially in the light of escalating climate change^1–3^. In the city of Madrid, Spain, the exposure to extreme temperatures has also been widely explored, with most studies focusing on the 2000s^4–7^. Heatwaves have been shown to significantly impact mortality, with certain socioeconomic factors (such as income level and housing) and chronic medical conditions increasing vulnerability^5,6,8^. Urban populations, particularly those in densely populated metropolitan areas, are at greater risk due to socioeconomic disadvantages^7^. Cold spells also contribute to increased mortality, especially from cardiovascular and respiratory-related causes. Research also indicates that the vulnerability to cold outweighs that of heat, with coldwaves consistently posing greater risks^4^. Nationwide, the impact of cold on mortality in Spain has significantly declined since the 1980 s, whereas the reduction in heat-related mortality risks has been much smaller^9,10^. Similarly, in Madrid, the effect of heat on mortality has slightly decreased over time, while the impact of cold saw a sharp decline until 2000. However, in the first decade of the 21 st century, cold-related mortality in Madrid showed a slight increase once again^11^. However, long-term studies on the evolution of adaptation to non-optimal temperatures remain limited, relying primarily on multidecadal time series^12–17^. This is likely due to the challenge of obtaining daily mortality counts and temperature data over such an extended period.

This study examines the daily temporal variations in optimum temperatures and temperature-attributable mortality fractions in Madrid from 1890 to 2020. Our aim here is not only to quantify the key temperature–mortality metrics over time, but also to comprehensively examine the potential drivers and underlying factors that may explain their observed changes. Over this 130-year period, the city has experienced significant demographic, social, and economic transformations, alongside a climatic shift characterized by rising average temperatures and decreasing annual precipitation^18,19^. Additionally, this period has been marked by a decline in mortality rates and an increase in life expectancy. This novel research underscores the unique use of over a century of daily data to examine how climatic and urban transformations interact over time, offering insights that were not addressed previously.

## Results

In total, we analyzed over 1.9 million deaths registered in the municipality of Madrid from 1890 to 2019. The summary statistics for temperature and population characteristics are presented in Table 1. The daily time series of total mortality counts and temperature are shown in the Supplementary Fig. 1.

**Table 1:** Descriptive statistics for temperature and population characteristics in the City of Madrid between the 1890 s and 2010s.

Decade	Temperature (^o^C)					Deaths	Population			Life expectancy^3^	
	Mean (sd)	*P*_1_	*P*_5_	*P*_95_	*P*_99_	Total	All ages^1^	< 10 (%)	> 60 (%)	At birth	At 60
1890–1899	12.7 (7.6)	0.1	2.7	26.4	28.5	165 950	470 283	16	6	23.2	10.7
1900–1909	12.3 (7.8)	0.2	2.6	26.7	29.0	158 553	539 835	16	6	27.6	10.8
1910–1919	11.9 (7.6)	0.1	2.7	26.2	28.4	158 950	599 807	17	7	38.0	13.0
1920–1929	12.8 (7.3)	1.4	3.3	25.6	27.4	159 603	750 896	16	7	37.3	11.7
1943–1951	13.6 (7.4)	0.3	3.4	26.2	28.4	92 978	1 618 435	15	6^2^	57.5	
1975–1989	13.5 (7.1)	2.0	4.6	26.4	28.6	239 799	3 188 297	14	16^2^	75.7	20.4
1990–1999	14.1 (7.4)	2.2	4.6	27.8	30.1	257 008	3 084 673	9	20	77.6	22.1
2000–2009	14.3 (7.5)	2.1	4.6	27.6	29.5	266 573	2 938 723	8	23	80.6	23.7
2010–2019	14.9 (7.8)	2.4	4.9	28.7	30.8	273 903	3 198 645	10	23	83.6	25.9

*sd* Standard deviation, *P*_1_ 1^th^ percentile of temperature distribution, *P*_5_5^th^ percentile of temperature distribution, *P*_95_ 95^th^ percentile of temperature distribution, *P*_99_ 99^th^ percentile of temperature distribution. ^1^Data on total population and population by age correspond to the official census estimates for 1887, 1900, 1910, 1920, 1950, 1981, 1991, 2001 and 2011. ^2^Share of population aged above 60 (60 not included), except: 1950 census where it is above 65, 65 y.o. not included, and 1981 census where it is 60 + and 60 y.o. are included. ^3^Life expectancy estimates for 1950, own estimations based on provincial level data. Estimates for 1981 onwards were provided by the National Institute of Statistics of Spain https://www.ine.es/ at the provincial level. Data on life expectancy for census years 1900 to 1920 originate from Dopico Gutiérrez del Arroyo, F., & Reher Sullivan, D. S. (1998)^20^. For census year 1887, data on life expectancy were kindly provided by Alberto Sanz Gimeno^21^.

**Fig. 1:**
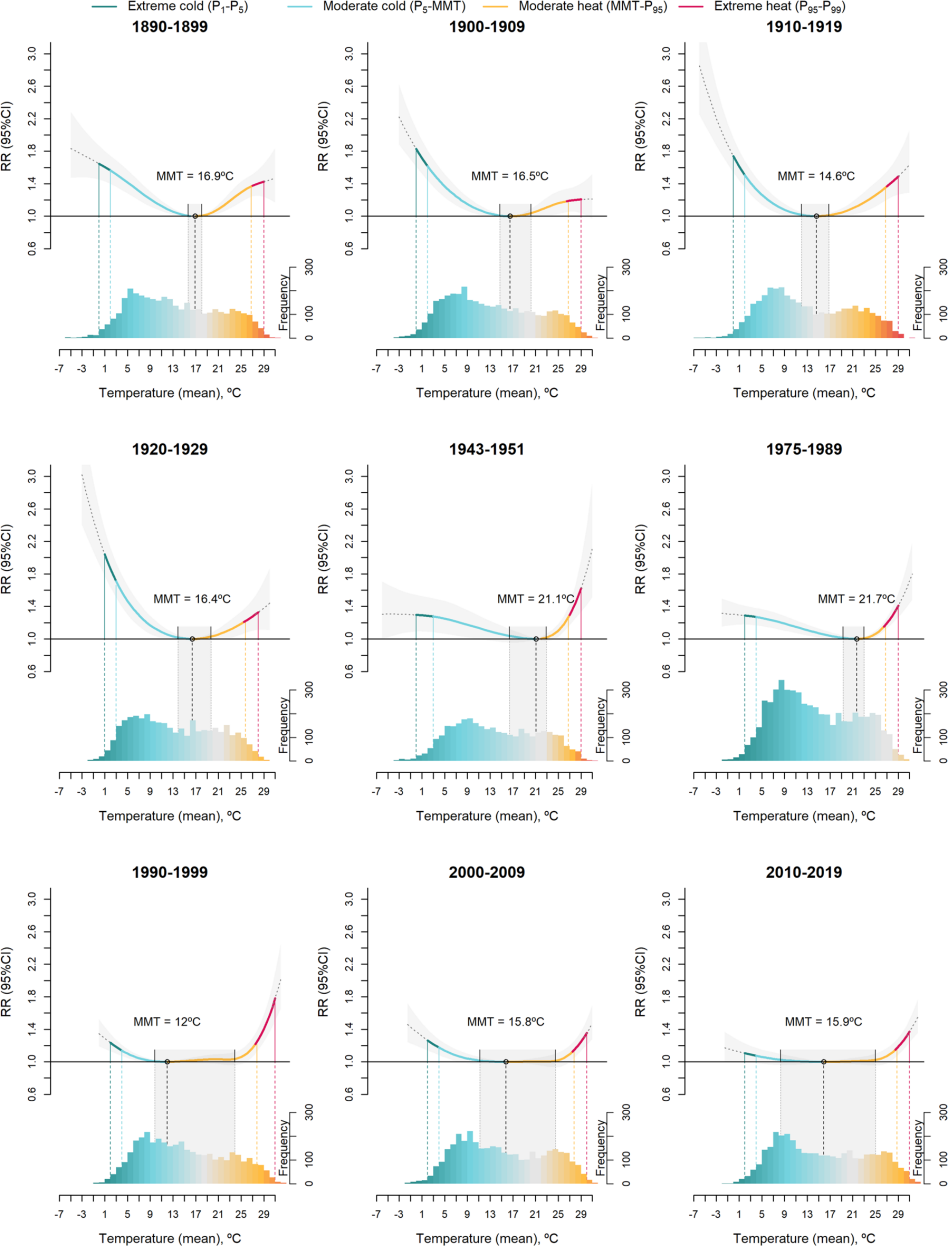
Overall cumulative temperature-mortality associations in Madrid between 1890 s and 2010 s, by decade.

The average temperature experienced a steady increase (+ 2.2 °C), while the population has grown by 6.8 times since the 1890s. The overall cumulative temperature-mortality associations exhibited a changing pattern throughout the study period, with the Exposure-Response (E–R) curve reshaping from a V-shape in the early decades to a U-shape in the later decades (Fig. 1). The minimum mortality temperature (MMT, the temperature at which mortality risk is lowest, statistically derived using Distributed Lag Non-Linear Models (DLNM) to capture the delayed and non-linear effects of temperature on mortality) shifted from 16.7 °C (95% CI=[15.2, 18.2]) in 1890–1899 to 13.8 °C (95% CI=[6.0, 19.7]) in 2010–2019. While the MMT estimates did not follow a consistent trend across decades, the confidence intervals around the MMT estimates expanded substantially over time, and the E–R curves become notably flatter near their minima. Since the 1980 s, there has been a marked warming of summer temperatures, alongside a gradual rise in winter temperatures that were notably colder during the first half of the 20th century. Until the late 1920 s, the cold effect on mortality was more pronounced, while in the most recent decades it has been significantly reduced. The heat effect also experienced a reduction, but on a much smaller scale compared to the cold. The impact of extreme heat remained practically unchanged.

**Fig. 2:**
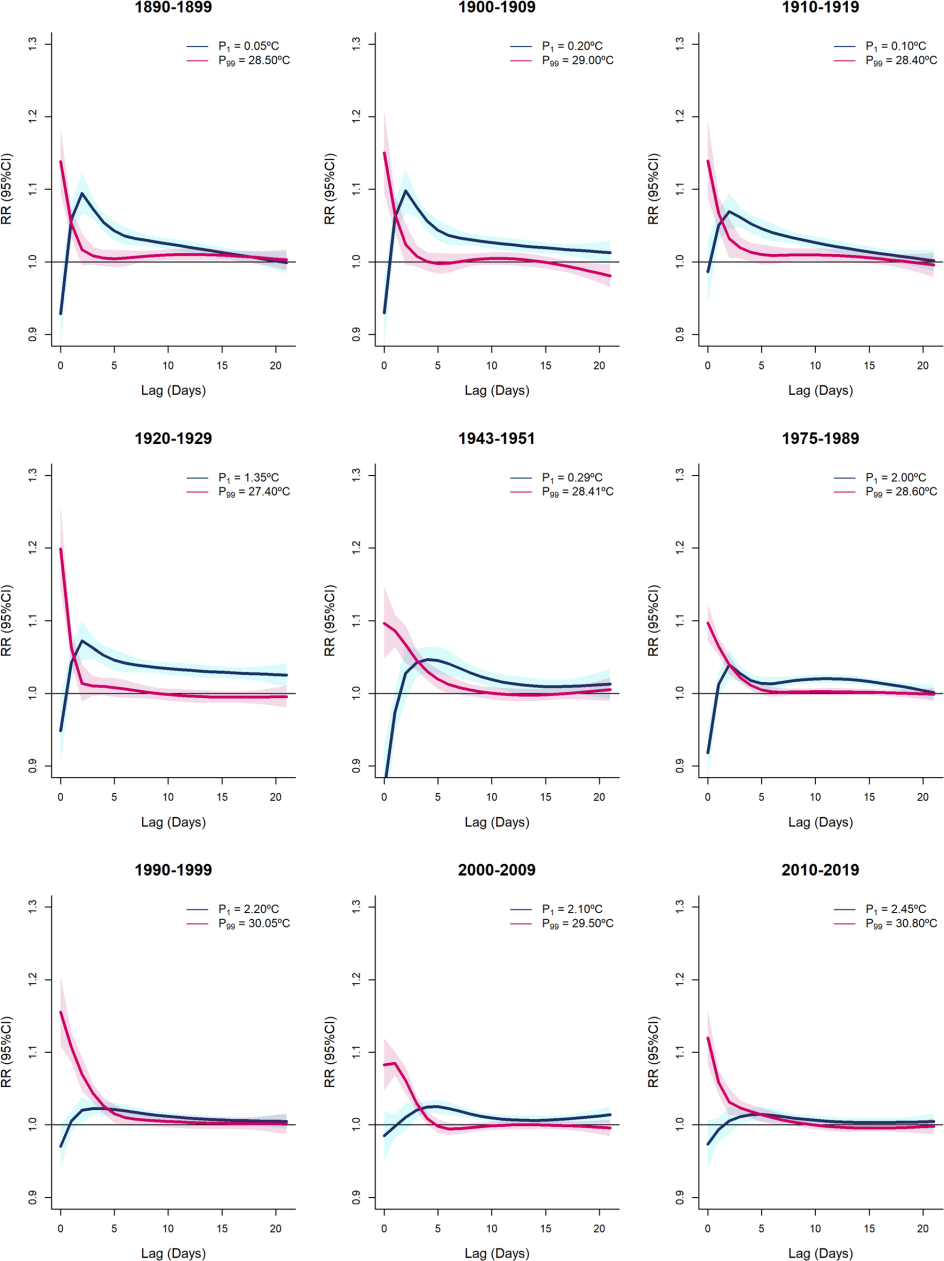
Lagged relative risks at percentiles P_1_ and P_99_ relative to its corresponding MMT between the 1890 s and 2010s.

The decade-specific lagged effect of cold gradually decreased throughout the study period, exhibiting larger and longer effects in the early decades and shorter and smaller effects in the later decades (Fig. 2). In contrast, the lagged effect of heat exhibited a more consistent pattern, showing a higher mortality risk on the same day of the exposure and lasting for a few days afterward, with the effect being more pronounced at the beginning of the 20^th^ century.

When comparing the initial (1890–1899) and final (2010–2019) periods, the attributable fraction of cold-related mortality experienced a substantial 7-fold reduction for extreme cold (from 2.27%, 95% CI=[1.74%−2.79%] to 0.33%, 95% CI=[−0.06%−0.70%]) and a 10-fold reduction for moderate cold (from 10.85%, 95% CI=[7.51%−14.24%] to 1.02%, 95% CI=[−1.47%−3.69%]). Heat-related mortality declined less significantly, with reductions of 1.5-fold (from 1.23%, 95% CI=[0.85%−1.60%] to 0.84%, 95% CI=[0.51%−1.13%]) and 3-fold (from 3.15%, 95% CI=[1.80%−4.42%] to 0.96%, 95% CI=[−0.87%−2.78%])) for extreme and moderate heat, respectively (Table 2).

**Table 2:** Mortality temperature (MMT), minimum mortality temperature percentile (MMTP), and attributable mortality fractions in Madrid between the 1890 s and 2010s.

Decade	MMT (ºC)	MMTP (%)	Mortality attributable fraction (%)			
			Extreme cold	Moderate cold	Moderate heat	Extreme heat
1890–1899	16.80 (15.70–18.00)	65.50 (61.70–69.50)	2.27 (1.74–2.79)	10.85 (7.51–14.24)	3.15 (1.80–4.42)	1.23 (0.85–1.60)
1900–1909	16.50 (14.70–20.60)	65.30 (58.60–77.70)	2.46 (1.96–2.98)	9.97 (6.39–13.44)	1.99 (0.20–3.67)	0.72 (0.25–1.20)
1910–1919	14.60 (12.10–16.80)	59.40 (50.80–65.90)	2.12 (1.65–2.57)	6.91 (3.34–10.26)	2.99 (1.31–4.74)	1.20 (0.75–1.66)
1920–1929	16.40 (13.90–19.90)	62.90 (53.90–75.70)	2.78 (2.19–3.36)	10.86 (6.60–15.01.60.01)	1.81 (0.20–3.27)	0.86 (0.39–1.33)
1943–1951	21.10 (16.50–22.90)	77.40 (61.10–84.10)	1.34 (0.41–2.15)	8.59 (1.03–15.29)	0.80 (0.11–1.43)	1.01 (0.66–1.34)
1975–1989	21.70 (19.20–23.00)	79.00 (70.70–83.40)	1.18 (0.78–1.60)	8.60 (5.00–12.22.00.22)	0.58 (0.23–0.93)	0.81 (0.57–1.03)
1990–1999	12.00 (9.80–23.90)	40.90 (29.80–83.80)	0.69 (0.40–0.99)	1.15 (0.26–2.01)	1.72 (−0.53-3.77)	1.27 (0.85–1.69)
2000–2009	15.80 (11.30–24.50)	55.80 (37.20–84.30)	0.80 (0.41–1.15)	2.12 (−0.41-4.29)	0.64 (−1.19-2.46)	0.72 (0.39–1.08)
2010–2019	15.90 (8.20–25.10)	53.20 (19.80–83.20)	0.33 (−0.06-0.70)	1.02 (−1.47-3.69)	0.96 (−0.87-2.78)	0.84 (0.51–1.13)

Moderate cold (P_5_ – MMT), Extreme cold (P_1_– P_5_), Moderate heat (MMT – P_95_), Extreme heat (P_95_ – P_99_).

**Fig. 3:**
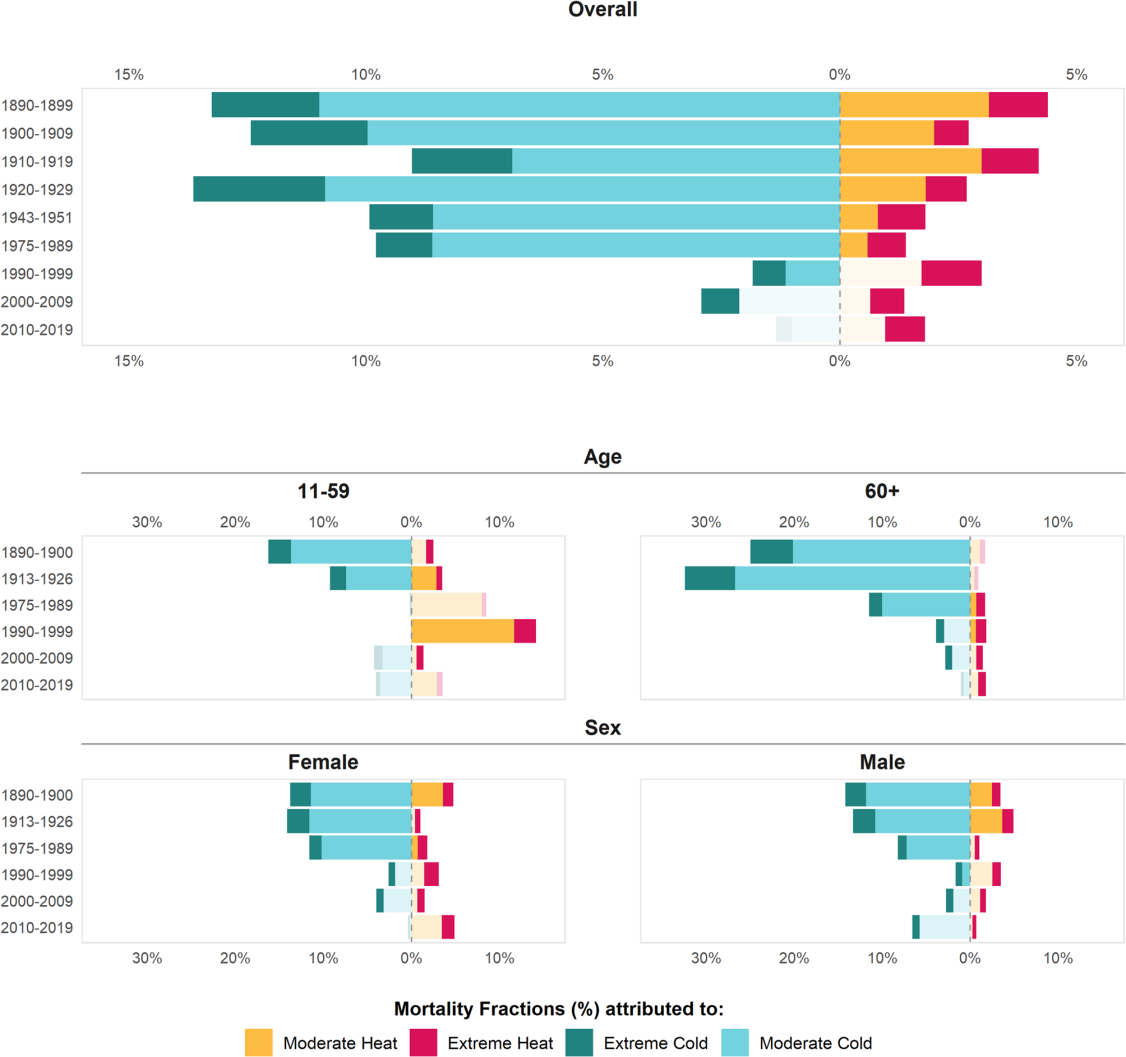
Attributable mortality fractions (%) in Madrid by decade, age- and sex- group. Moderate cold (P_5_ – MMT), Extreme cold (P_1_ – P_5_), Moderate heat (MMT – P_95_), Extreme heat (P_95_ – P_99_). The transparency indicates non-significate estimates at CI95%.

The reduction in the attributable fraction of cold-related mortality was also substantial for both males and females until the early 1990s (Supplementary Table 1). In the subsequent decades, only extreme cold remained significant in contributing to the mortality burden, particularly for males (0.83%, 95% CI=[0.06%−1.49%] in 2010–2019), while moderate low temperatures showed no significant impact for either sex. The reduction in heat-attributable mortality was more pronounced in males from the 1980s onward, with only the extreme heat fraction remaining significant (0.44%, 95% CI=[0.14%−0.74%] in 2010–2019). In females, extreme heat showed a slight increase in the last decade (from maximum of 1.65%, 95% CI=[1.10%−2.19%] in 1990–1999 down to 0.84%, 95% CI=[0.46%−1.17%] in 2000–2009 to 1.42%, 95% CI=[0.89%−1.90%] in 2010–2019) (Fig. 3).

When considering age, the population over 60 years old was more susceptible to both heat and cold exposure than those aged 11–59 years (Supplementary Fig. 2, Supplementary Table 1). The moderate cold resulted in the highest impacts on the elderly population in the 1913–1926, reaching 26.7%, 95% CI=[19.1%−33.7%]. Half a century later (1975–1989) the moderate cold was still responsible for a significant proportion of 10%, 95% CI=[5.8%−14.1%] of all deaths among the 60+. Later on, these effects disappeared for the moderate cold while the impact for extreme cold remained low but present until 2000–2009 for the elderly. Interestingly, the effects of heat for the elderly were not significant in the earlier periods, while since 1975 the extreme heat exhibited a rather stable and negative effect across all population age groups.

## Discussion

### A century of change: unpacking the driving forces

The period covered in this study spans more than a century — a timeframe marked by profound transformations in climate, urban environments, demographic structure, and socioeconomic conditions. The combination of these factors not only altered the physical environment but also redefined the ways populations interact with climate-related stressors such as extreme temperatures. Any attempt to model temperature–mortality associations over such an extended period at any location must reckon with this complexity. Understanding the evolution of this association over more than a century requires a holistic perspective that considers the substantial transformations in both the population and the environment.

Our 30-year moving windows analysis based on the Madrid´s climate normals suggests an ongoing transition from a hot-summer Mediterranean climate, characterized by higher precipitation and greater ecological productivity, to a dry and extreme cold semi-arid climate since the late 1980s (Supplementary Fig. 3). This shift is primarily attributed to a pronounced and sustained increase in mean temperatures throughout the year, with the most significant warming observed during the summer months, and overall notable decrease in the frequency of extremely cold days. This progressive warming has been accompanied by a gradual decline in annual precipitation, particularly in spring and early autumn, contributing to an increasingly arid regime. This transition is consistent with broader picture across the Iberian Peninsula and the Mediterranean region^18,19,22^. However, it is worth noting that the precipitation trends across Spain are highly variable and often not statistically significant at the national level, due to spatial heterogeneity and the complex nature of Mediterranean climates^23,24^.

The observed changes in the temperature–mortality relationship, particularly the reduction in risks associated with cold exposure, are unlikely to be driven solely by winter warming. Instead, they likely reflect broader societal and structural transformations that have progressively shaped the vulnerability profile of Madrid´s population — either dampening or amplifying the health impacts of temperature extremes. In the late 19^th^ and early 20^th^ centuries, the city of Madrid experienced a demographic transition, characterized by declining mortality rates and a gradual reduction in fertility, shifting from a high-mortality, high-fertility regime to a more stable population structure. This process was temporarily disrupted by major epidemiological shocks, including the Russian flu (1889–1890) and the Spanish flu (1918–1919), which slowed mortality declines and reshaped public health responses and population resilience. Over the 20th century, improvements in living conditions and rising life expectancy reduced vulnerability to infectious diseases, but aging population and intensive urbanization introduced new susceptibilities.

Vulnerability to temperature extremes is a complex concept shaped by a range of factors related both to individual characteristics—such as age, underlying health status and socioeconomic position—and to contextual conditions, including the physical and social environment. The development of the infrastructure, urbanization, and wider availability of the healthcare services have changed the population’s adaptive capacity, potentially mitigating temperature-related risks. At the same time, the physical environment has undergone changes, including modifications in land use and vegetation cover, which can alter the local climate and either amplify or attenuate the impact of temperature fluctuations on mortality. Despite these insights, a complete understanding of all the factors driving changes in temperature-related mortality remains elusive. One way to critically examine this issue is through the urban paradox—the observation that while urbanization generally improves living standards and healthcare access, it also introduces new vulnerabilities, particularly through increased heat retention, air pollution, and socio-spatial inequalities.

This paradox is particularly relevant for Madrid, which, over the past 130 years, has expanded from a city of 470,283 inhabitants in 1887 to a metropolitan hub exceeding 3 million in 2024. Such rapid urban growth has had profound implications for population health and exposure to environmental risks. In the early 20th century, Madrid’s population growth outpaced the development of its infrastructure, leading to overcrowding, inadequate sanitation, and poor housing conditions, particularly in peripheral areas. While Spain as a whole lagged behind other European nations in reducing mortality rates, Madrid saw a more accelerated decline, largely due to advancements in water sanitation^25^, public health policies, and healthcare accessibility. Between 1880 and 1930, central Madrid underwent a significant transformation, evolving into a modern capital^26^ with major investments in sanitation systems, new architectural developments, national institutions, education, and commercial infrastructure. However, stark socio-spatial inequalities persisted, with peripheral districts experiencing lower living standards and greater exposure to environmental hazards^27,28^. By the mid-to-late 20th century, slums were progressively eradicated, improving housing conditions and reducing exposure to extreme cold during winter months. From mid 20th century central heating systems began to be installed in new buildings, and many older structures were retrofitted. These systems, powered initially by coal and wood and later on by oil or gas, provided a more efficient and consistent source of heating, reducing mortality risks associated with cold exposure, particularly among the elderly.

Similarly, the increasing use of air conditioning in Madrid has played a crucial role in modifying heat-related mortality patterns. While air conditioning started to become more widespread in the 1970 s, its availability in residential properties surged in the 1990 s and 2000 s as technology improved and prices became more affordable. By 2012 however, 26.6% of households in the Madrid region lacked comfortable indoor temperatures during the summer, a share that increased to 37.7% in 2023 (2012 and 2023 Living Conditions Survey, INE). This contrast may reflect the influence of socioeconomic conditions, as periods of economic or energy strain may lead households to limit the use of cooling systems, even when they are available. The critical role of air conditioning was demonstrated in the 3HEAT study^29^, which considered the effects of power outages. Energy or price crises may limit its use among socially vulnerable populations—particularly the elderly—due to the affordability constraints. The adoption and widespread uptake of air conditioning among households, combined with enhanced public health measures against heatwaves, has contributed to a slight reduction of heat-related mortality in Madrid in recent decades compared to the late 19th and early 20th centuries. However, the benefits of air conditioning are still not equally distributed, and socio-economic disparities continue to condition differential vulnerability to heat across Madrid’s population in the modern times^8^.

Overall, the long-term changes in the temperature-mortality relationship in Madrid reflect a complex interplay of demographic shifts, urban development, infrastructural advancements, and environmental changes. While improvements in housing, heating, and cooling systems have reduced vulnerability to extreme temperatures, emerging challenges—such as aging population and increasing heatwave frequency—continue to influence mortality patterns, requiring continuous adaptation and policy interventions.

### Rationale for modeling strategy and implications for interpretation

Understanding long-term changes in temperature-related mortality requires careful consideration of both methodological choices and how results are interpreted. One key aspect is how population dynamics are accounted for, particularly when using death counts without a population offset or equivalent demographic adjustment. Many DLNM-based time-series studies, as can be seen in many publications produced by the Multi-Country Multi-City Collaborative Research Network (MCC, an international collaboration of research teams working on a program aiming to produce epidemiological evidence on associations between environmental stressors, climate, and health; available at https://mccstudy.lshtm.ac.uk/publications/), analyze death counts directly, assuming the offset term (log-population) is constant or implicitly controlled through time spline terms. This is partly justified in single-location time-series studies where daily or short-term fluctuations in population size are negligible, and where smooth time trends account for broader demographic shifts. Our models include long-term and seasonal trends via splines on time. These smooth functions help capture slow changes in mortality risk that may reflect changes in demographics, healthcare, or other time-varying confounders—including population size and structure. We also stratified by demographic groups (age and sex), and within each stratum, MMT and attributable fractions (AFs) were estimated independently. Though population denominators changed over time, the temporal smoothers still help absorb major secular trends (e.g. population growth, aging, changes in baseline mortality risk), and group-based analyses allow us to describe how temperature sensitivity may differ between demographic segments—even without population-standardized rates. While not a perfect proxy, this adjustment mitigates some of the structural confounding from population dynamics. One way to address this limitation and improve the comparability over time is to explicitly incorporate time-varying population denominators and structure (e.g., age-standardized rates and offset-adjusted models) to assess their impact on temperature–mortality associations. Given that the primary objective of this study is not methodological innovation, we opted for a parsimonious modeling approach, avoiding additional complexity. Instead, we focused on deriving a full set of classical metrics typically estimated from DLNMs, in order to facilitate interpretation of long-term trends.

In this context, we argue that the MMT should not be interpreted as a standalone indicator of change. Misinterpretation of the MMT trends commonly arises when its temporal fluctuations are assessed without considering the overall shape of the E–R curve, the width and steepness of the response around MMT, and importantly, the confidence intervals associated with MMT estimates. This flattening of the curve and widening of the confidence intervals suggest not simply increased statistical noise, but potentially greater tolerance or resilience of the population to a wider range of temperatures — a phenomenon that may reflect behavioral, infrastructural, or physiological adaptation. Another plausible contributing factor is the increasing variability in daily temperatures in recent decades, which affects both the estimation precision and the curve shape.

Finally, examining changes in attributable fractions alongside shifts in the lag structure of heat- and cold-related mortality responses also helps to establish a clearer understanding of temperature–mortality dynamics. Over the years, and especially in the first decades of the observation, the cold-attributable mortality fraction in Madrid has substantially declined across various demographic groups. In contrast, the extreme heat-attributable fraction pattern has not followed the same downward trend. Since the early 2000 s, heat-attributable mortality fraction has remained stable at approximately 1%, with a slight increase in recent years, potentially linked to a rising incidence of heatwaves and an aging population. Furthermore, the delayed effects of heat and cold exposure manifest differently^30–32^: cold exposure is associated with prolonged delayed effects, extending up to three weeks, likely due to the gradual development of respiratory conditions. Heat exposure, on the other hand, has more immediate impacts, potentially due to acute cardiovascular or cerebrovascular events. The overall pattern of delayed effects has remained consistent over time. In the case of cold exposure, the initial peak in mortality during the first few days was particularly pronounced in the earlier decades. However, as overall risk declined over time, the effect became more stable, though still present, throughout the three weeks following exposure. This reflects changes in cold resistance over time and the different age pattern of this curve. At the turn of the 20th century, mortality during low temperatures was particularly high among children, especially those under one year old, as seen in neonatal mortality in Italy^33^, as well as among the elderly. In earlier periods of our study, children exhibited an elevated risk for up to two weeks, though it declined significantly after the first week, whereas older individuals experienced a persistently high risk beyond three weeks. In more recent periods, this pattern has shifted, with the elevated risk among older individuals significantly decreasing after 10 days, while in children, although the risk continues to diminish after one week, the results are not statistically significant (Supplementary Fig. 4). The observed changes in the effects of cold on mortality and the decline in lag effects over time result from a combination of factors, including advancements in medical treatment, improved housing and climate control, and overall enhancements in population health and socioeconomic status. For heat exposure, the pattern has shown little variation over the years, with a sharp peak on the day of exposure, followed by a rapid decline, reaching a minimum within approximately one week.

### Limitations and opportunities

While this study benefits from a long-term historical perspective, it also comes with inherent limitations. Many parallel historical changes that may have influenced temperature-related mortality are poorly documented, limiting the ability to fully capture the complexity of interactions between climate, urbanization, and population health. One key limitation is the lack of individual or household-level data on heating and air conditioning availability. The only available information on these facilities comes from aggregated estimates for a limited number of years—at the regional level (2007, Living Conditions Survey) and at the city level (1991, 2001 and 2011 Population and Housing Census). Although previous studies have suggested an association between increased air conditioning use and reduced heat-related mortality in Spain, none have directly examined the presence or absence of air conditioning at the household level, making it difficult to quantify its precise role in modifying temperature-related risks^34–36^.

Another limitation is the use of all-cause mortality data, which, while comprehensive, does not differentiate between physiological pathways leading to death due to temperature exposure. Cold and heat affect mortality through distinct mechanisms: extreme temperatures impair thermoregulation, leading to cardiovascular events and the exacerbation of respiratory conditions. Heat exposure has been linked to increased mortality from nervous system disorders, diabetes, and kidney diseases^37^, while cold exposure is known to aggravate asthma and chronic obstructive pulmonary disease^38,39^. In this study, cause-specific mortality data were only available for 1890–1900 and 1913–1927, leaving significant data gaps for other periods. Future research should aim to refine these findings by incorporating more detailed cause-of-death analyses.

The location of the meteorological station used for temperature data presents an important constraint. Initially situated in a park on the outskirts of Madrid in the early 20th century, with urban expansion, the station—still in the same park—is now surrounded by dense development and heavy traffic in a more central part of the city. Although the distance from the urban core was in close proximity (1–2 km) even in the early decades, the surrounding environment has changed dramatically over time. This transformation—from a relatively peripheral green space to a highly urbanized zone—likely affected temperature measurements through the intensification of the urban heat island (UHI) effect, changes in surface composition and traffic-related emissions^40,41^. As the city expanded, the built environment contributed to increased temperatures through heat absorption by buildings, reduced green spaces, and anthropogenic heat sources. While this UHI phenomenon has become more pronounced over time, separating the effects of climate change from localized urban warming remains challenging^42^. Moreover, air pollution trends have evolved, with declining concentrations of sulfur dioxide and carbon monoxide but stable levels of nitrogen dioxide and particulate matter since 1990^43^. Notably, ozone concentrations increased by up to 40% between 2007 and 2014, potentially modifying the interactions between temperature and health outcomes^44,45^. These evolving local conditions introduce potential exposure misclassification, particularly in earlier periods, and may complicate the interpretation of long-term trends in temperature–mortality associations. We recognize that due to the design this study does not account for the city growth, increased automobile use, or changing pollution levels, all of which could influence local temperature and environmental conditions, and affect the severity of adverse health outcomes.

Finally, this study did not control for humidity, which is a relevant effect modifier of the relationship between temperature and mortality. At high temperatures, elevated humidity reduces the efficiency of evaporative heat loss, thereby increasing thermal strain. Some studies, however, highlight the absence of a positive association of humidity with mortality in the summer^46^. In cold conditions, humidity may affect heat exchange and thermal comfort, although its influence on cold-related mortality is less consistently demonstrated. While the omission of humidity may have introduced some residual confounding, adjusting for humidity as a standalone covariate may not adequately capture its physiological relevance. Composite thermal indices—such as Apparent Temperature, Humidex, or Wet Bulb Globe Temperature (WBGT)—integrate temperature and humidity to better reflect perceived thermal stress. Incorporating such indices represents a valuable direction for our future research aimed at refining the assessment of temperature-related health risks.

## Conclusion

The long-term decline in temperature-related mortality in the city of Madrid was largely driven by a marked reduction in cold-attributable mortality, as quantified across the study period. This reduction was accompanied by clear changes in the temperature–mortality relationship, including lower sensitivity to cold exposure and redistribution of seasonal mortality patterns. At the same time, the decreasing frequency of extremely cold days and rising average temperatures have further reduced the overall burden associated with cold exposure. These long-term changes occurred alongside major demographic, environmental, and technological transformations in Madrid over more than a century. Although this study does not explicitly assess the role of specific drivers, the reduction in cold-attributable mortality is consistent with broader improvements in living conditions, healthcare, urban infrastructure, and access to heating over the study period.

In contrast, heat-related mortality has followed a more complex trajectory. Although the impact of moderate heat has declined over time, the effect of extreme heat has remained relatively stable, with a slight increase in recent decades. This pattern highlights the acute nature of heat stress, particularly among individuals of advanced age as shown in our study. Over recent decades, Madrid has experienced marked population aging, with a steady increase in life expectancy, declining fertility, and a considerable increase in the proportion of adults aged 85 years and older. In this context, continued public health interventions—such as heat prevention campaigns targeting older adults and other high-risk groups—remain essential. Moreover, potential future energy crises represent an additional concern, as increasing economic vulnerability could amplify mortality risks during both cold and heat extremes.

Overall, this study provides a long-term historical perspective on the evolving temperature–mortality relationship in the city of Madrid over more than a century. The findings illustrate the dual impact of rising temperatures, which are associated with both mitigation of cold-related mortality and persistent vulnerability to extreme heat. Future research will integrate more detailed population-level data to further investigate adaptive mechanisms across different demographic groups, providing a deeper understanding of resilience in the face of changing climatic conditions.

## Methods

### Data collection

#### Mortality data

Daily all-cause mortality statistics by age and sex for the city of Madrid cover the period from 1888 to 2019 and were obtained from the yearly books and civil register of the city. The compilation of the dataset for the beginning of the study period was performed manually, while for the later decades the data were provided in the digital form. Total daily death counts cover 8 complete naturals decades (1890–1929, 1980–2019), a period of 9 years between 1943 and 1951, and a period of 5 years between 1975 and 1989. Age- and sex-specific mortality data are available for two discontinuous periods in the beginning of the 20th century (1890–1900 and 1913–1926) and for a larger period between 1975 and 2019.

#### Temperature data

The daily average air temperatures were provided by the State Meteorological Agency (AEMET) for the period between 1890 and 2019 for the station of the Retiro Park (40°24’43’’ N, 3°40’41’’ W). These values were initially calculated as the arithmetic mean of continuous temperature measurements recorded throughout the 24-hour period. The measurement station is located within the city park of Retiro with the surface area around 300 hectares in the historical center of Madrid on flat and horizontal ground. The exact position and surroundings of the station have changed in the course of time due to urban development of the capital (Supplementary Fig. 5). The time-series of the measurements contain missing dates during the period of the Spanish Civil war (51 days in 1936, 61 days in 1937 and 33 days in 1939) and a few single missing dates in other years. To fill these gaps, we used the blended data from the European Climate Assessment & Dataset (ECA&D)^41^.

### Statistical analysis

We modelled the temperature-mortality association for each periods using time-series regression with distributed lag non-linear models^47^. A standard time-series quasi-Poisson regression was applied to derive an overall, age- and sex-specific temperature-mortality association, reported as relative risks (RR) where minimum mortality temperature (MMT) acts as a reference temperature for calculating the RR. The decadal model could be formalized as follows:

$$Y{\text{ }}\sim {\text{ }}Poisson(\mu )$$

$$\log E(\mu ) = \alpha + cb + ns\left( {time,10df \times year} \right) + DoW$$

where Y denotes the observed daily death count in the city of Madrid, α is the intercept, cb is the cross-basis matrix for the two dimensions of predictor and lags produced by distributed lag non-linear model, ns stands for the B-spline basis matrix for a natural cubic spline with 10 degrees of freedom per year to control for seasonal and long-term trends, and DoW represents an indicator of the day of the week.

We controlled for long-term trend and seasonal cycles using a natural cubic spline of time with 10 degrees of freedom per year and indicator variables for the day of the week. The temperature-response was modelled with a natural cubic spline with three internal knots placed at the 10^th^, 75^th^, and 90^th^ percentiles of the temperature distribution, and the lag-response, up to 21 days, with a natural cubic spline with three internal knots placed at equally spaced values in the log scale. The parameter choices were tested through sensitivity analyses (Supplementary Table 2).

The overall analysis covers nine time periods, while the age- and sex-specific analysis was performed for six discontinuous decades as described earlier. Moreover, the age-specific analysis was conducted for two age groups: 11–59 years and older than 60 years. From the estimated curve representing the overall cumulative exposure-response (the net effect across lags), we identified the minimum mortality temperature (MMT) jointly with an approximate parametric bootstrap estimator of its confidence interval and standard error^48^. We also calculated the MMT percentile (MMTP), defined as the percentile of the temperature distribution corresponding to the MMT. We estimated the attributable fractions of mortality^49^ due to moderate cold and heat, defined at the 5^th^ and 95^th^ percentiles (P_5_ and P_95_) of the temperature distribution, compared to the MMT of each period. We also estimated the attributable fractions due to extreme temperatures by comparing the 1st and 99^th^ percentiles (P_1_ and P_99_) and the P_5_ and P_95_, respectively.

### Statistical software

The statistical analyses were conducted in R software (version 4.4.0) using functions from the *dlnm* package^50^.

## Supplementary Information

Below is the link to the electronic supplementary material.

Supplementary Material 1

Supplementary Material 2

Supplementary Material 3

Supplementary Material 4

Supplementary Material 5

Supplementary Material 6

Supplementary Material 7

Supplementary Material 8

Supplementary Material 9

## Data Availability

The meteorological observational data from 1920 onwards can be freely obtained from the European Climate Assessment and Dataset ([www.ecad.eu](http:/www.ecad.eu)). The meteorological data for earlier years can be obtained via official request to the State Meteorological Agency of Spain. Mortality data from 1975 onward can be requested from the National Institute of Statistics of Spain (INE), while data for earlier years can be obtained from the authors upon request. Due to INE privacy regulations and the conditions of the data supply contract, the mortality data used in this study cannot be made publicly available. Data requests should be addressed to [diego.ramiro@cchs.csic.es](mailto: diego.ramiro@cchs.csic.es).
