# Supplementary material for: Exploring 130 years of temperature-related mortality in the city of Madrid
Source: Sci Rep. 2026 Feb 25;16:7641. doi: 10.1038/s41598-026-38595-4 (PMC12936060; doi:10.1038/s41598-026-38595-4)

**Supplementary Table 1.** Attributable mortality fractions by age- and sex- group in Madrid between the 1890s and 2010s.

| **Decade** |  | **Mortality attributable fractions (%)** | | | |
| --- | --- | --- | --- | --- | --- |
|  |  | **Extreme cold** | **Moderate cold** | **Moderate heat** | **Extreme heat** |
| **1890-1900** | 11-59 | 2.57 (1.81- 3.25) | 13.69 (7.54-19.27) | 1.66 (-0.15- 3.40) | 0.81 (0.28- 1.35) |
|  | 60+ | 4.81 (3.69- 5.93) | 20.17 (11.98-27.00) | 1.09 (-0.93- 3.06) | 0.56 (-0.10- 1.20) |
|  | Female | 2.35 (1.76- 3.02) | 11.43 (6.67-15.63) | 3.56 (1.60- 5.36) | 1.18 (0.70- 1.62) |
|  | Male | 2.33 (1.71- 2.93) | 11.85 (7.32-16.01) | 2.45 (0.76- 3.98) | 0.96 (0.50- 1.40) |
| **1913-1926** | 11-59 | 1.80 (1.06- 2.45) | 7.45 (1.77-12.15) | 2.81 (0.12- 4.94) | 0.68 (0.04- 1.23) |
|  | 60+ | 5.71 (4.65- 6.75) | 26.72 (19.14-33.66) | 0.48 (-0.73- 1.55) | 0.42 (-0.09- 0.87) |
|  | Female | 2.53 (1.62- 3.35) | 11.60 (4.11-18.53) | 0.38 (-0.10- 0.84) | 0.64 (0.26- 1.01) |
|  | Male | 2.51 (1.87- 3.13) | 10.82 (6.26-15.01) | 3.64 (1.68- 5.40) | 1.25 (0.71- 1.77) |
| **1975-1989** | 11-59 | 0.21 (-0.01- 0.45) | 0.00 (0.00- 0.00) | 8.00 (-4.46-18.89) | 0.46 (-0.66- 1.42) |
|  | 60+ | 1.51 (1.04- 1.99) | 10.00 (5.82-14.10) | 0.68 (0.33- 1.03) | 1.00 (0.77- 1.24) |
|  | Female | 1.39 (0.81- 1.92) | 10.22 (4.50-15.63) | 0.71 (0.30- 1.11) | 1.09 (0.80- 1.40) |
|  | Male | 1.01 (0.43- 1.52) | 7.23 (2.23-12.03) | 0.48 (-0.24- 1.14) | 0.54 (0.22- 0.87) |
| **1990-1999** | 11-59 | 0.07 (-0.35- 0.46) | 0.01 (-0.14- 0.16) | 11.64 (2.88-19.04) | 2.49 (1.36- 3.54) |
|  | 60+ | 0.92 (0.37- 1.40) | 2.97 (-2.18- 7.93) | 0.64 (0.32- 0.98) | 1.18 (0.83- 1.52) |
|  | Female | 0.71 (0.20- 1.25) | 1.89 (-1.00- 4.62) | 1.43 (-0.76- 3.32) | 1.65 (1.10- 2.19) |
|  | Male | 0.73 (0.33- 1.09) | 0.95 (0.20- 1.76) | 2.52 (-0.76- 5.70) | 0.96 (0.30- 1.51) |
| **2000-2009** | 11-59 | 0.95 (-0.42- 2.13) | 3.30 (-14.22-16.76) | 0.55 (-0.28- 1.45) | 0.81 (0.02- 1.51) |
|  | 60+ | 0.78 (0.36- 1.18) | 2.08 (-0.41- 4.46) | 0.69 (-1.20- 2.57) | 0.73 (0.35- 1.09) |
|  | Female | 0.81 (0.11- 1.44) | 3.18 (-3.55- 9.74) | 0.63 (-0.04- 1.26) | 0.84 (0.46- 1.17) |
|  | Male | 0.85 (0.39- 1.30) | 1.92 (-0.28- 4.10) | 1.10 (-1.88- 3.69) | 0.66 (0.13- 1.16) |
| **2010-2019** | 11-59 | 0.50 (-0.87- 1.77) | 3.55 (-6.73-12.70) | 2.88 (-1.69- 6.98) | 0.64 (-0.44- 1.67) |
|  | 60+ | 0.32 (-0.04- 0.67) | 0.76 (-1.10- 2.55) | 0.89 (-1.41- 3.07) | 0.87 (0.49- 1.22) |
|  | Female | 0.16 (-0.22- 0.53) | 0.22 (-0.73- 1.07) | 3.44 (-0.73- 7.72) | 1.42 (0.89- 1.90) |
|  | Male | 0.83 (0.06- 1.49) | 5.74 (-2.12-12.70) | 0.23 (-0.11- 0.56) | 0.44 (0.14- 0.74) |

Moderate cold (P_5_ vs. MMT); Extreme cold (P_1_ vs. P_5_); Moderate heat (P_95_ vs MMT); Extreme heat (P_99_ vs. P_95_).

**Supplementary Table 2.** Sensitivity analysis on knots for temperature distribution, lag duration, and number of degrees of freedom (df).

|  |  |  | **1890-1899** | | **1900-1909** | | **1910-1919** | | **1920-1929** | | **1943-1951** | | **1975-1989** | | **1990-1999** | | **2000-2009** | | **2010-2019** | |
| --- | --- | --- | --- | --- | --- | --- | --- | --- | --- | --- | --- | --- | --- | --- | --- | --- | --- | --- | --- | --- |
| Knots | Lag | df | RR at 28.5ºC | MMT | RR at 29.0ºC | MMT | RR at 28.4ºC | MMT | RR at 27.4ºC | MMT | RR at 28.4ºC | MMT | RR at 28.6ºC | MMT | RR at 30.1ºC | MMT | RR at 29.5ºC | MMT | RR at 30.8ºC | MMT |
| 10,25,75,90 | 7 | 7 | 1.21 (1.13-1.30) | 16.4 | 1.27 (1.18-1.36) | 14.4 | 1.22 (1.15-1.30) | 19.5 | 1.30 (1.20-1.42) | 9.5 | 1.50 (1.39-1.62) | 19.5 | 1.31 (1.25-1.37) | 21.4 | 1.44 (1.37-1.51) | 22.4 | 1.33 (1.25-1.41) | 10.6 | 1.45 (1.37-1.54) | 9.5 |
| 10,25,75,90 | 7 | 10 | 1.26 (1.17-1.36) | 14.6 | 1.30 (1.21-1.40) | 15.6 | 1.34 (1.25-1.45) | 15.3 | 1.43 (1.32-1.54) | 15 | 1.53 (1.41-1.66) | 17.1 | 1.35 (1.28-1.41) | 16.5 | 1.48 (1.39-1.59) | 13.6 | 1.34 (1.26-1.42) | 14.3 | 1.41 (1.32-1.50) | 8.2 |
| 10,25,75,90 | 7 | 13 | 1.21 (1.12-1.31) | 14.8 | 1.32 (1.22-1.42) | 14.7 | 1.35 (1.24-1.47) | 13.9 | 1.49 (1.37-1.63) | 11.5 | 1.58 (1.44-1.72) | 16.2 | 1.36 (1.28-1.45) | 11.9 | 1.51 (1.40-1.62) | 12.8 | 1.33 (1.25-1.42) | 13 | 1.39 (1.30-1.49) | 10.5 |
| 10,25,75,90 | 14 | 7 | 1.24 (1.14-1.36) | 16.8 | 1.25 (1.13-1.38) | 12.8 | 1.16 (1.04-1.28) | 12 | 1.11 (0.99-1.25) | 9 | 1.49 (1.35-1.65) | 21.9 | 1.32 (1.24-1.40) | 23.2 | 1.41 (1.32-1.51) | 23.6 | 1.32 (1.21-1.43) | 9.8 | 1.45 (1.34-1.58) | 9.9 |
| 10,25,75,90 | 14 | 10 | 1.37 (1.24-1.51) | 15.1 | 1.33 (1.19-1.49) | 15.9 | 1.43 (1.28-1.58) | 14.7 | 1.35 (1.21-1.51) | 17 | 1.49 (1.33-1.68) | 18.9 | 1.34 (1.25-1.44) | 22 | 1.51 (1.37-1.67) | 12.8 | 1.32 (1.23-1.42) | 18.2 | 1.37 (1.28-1.47) | 17 |
| 10,25,75,90 | 14 | 13 | 1.32 (1.17-1.48) | 14.9 | 1.46 (1.28-1.66) | 13.3 | 1.48 (1.30-1.68) | 13.5 | 1.44 (1.26-1.64) | 13.4 | 1.57 (1.38-1.80) | 18.1 | 1.33 (1.24-1.44) | 22.8 | 1.59 (1.42-1.78) | 11.7 | 1.30 (1.18-1.43) | 13.6 | 1.34 (1.22-1.48) | 13.7 |
| 10,25,75,90 | 21 | 7 | 1.22 (1.09-1.36) | 16.4 | 1.13 (0.99-1.29) | 12.6 | 1.06 (0.92-1.22) | 10.1 | 1.17 (1.06-1.30) | 30 | 1.55 (1.36-1.76) | 22.8 | 1.33 (1.23-1.44) | 23.7 | 1.39 (1.28-1.50) | 24.1 | 1.28 (1.14-1.42) | 9.1 | 1.43 (1.28-1.60) | 9.8 |
| 10,25,75,90 | 21 | 10 | 1.41 (1.24-1.60) | 15.9 | 1.21 (1.04-1.41) | 15.2 | 1.44 (1.25-1.66) | 15.8 | 1.30 (1.12-1.52) | 17.7 | 1.50 (1.28-1.76) | 19.8 | 1.38 (1.26-1.52) | 22.9 | 1.60 (1.38-1.84) | 11.8 | 1.30 (1.18-1.43) | 20.5 | 1.36 (1.24-1.49) | 20.7 |
| 10,25,75,90 | 21 | 13 | 1.42 (1.20-1.68) | 15.5 | 1.48 (1.21-1.81) | 11.8 | 1.53 (1.27-1.85) | 15.1 | 1.46 (1.22-1.74) | 16.1 | 1.64 (1.35-1.99) | 18.9 | 1.39 (1.25-1.55) | 23.6 | 1.85 (1.55-2.20) | 10.3 | 1.23 (1.10-1.39) | 18.5 | 1.28 (1.13-1.44) | 19.3 |
| 10,50,90 | 7 | 7 | 1.21 (1.13-1.29) | 17.3 | 1.26 (1.18-1.35) | 17.1 | 1.23 (1.15-1.31) | 19.4 | 1.27 (1.17-1.38) | 8.5 | 1.50 (1.39-1.62) | 19.7 | 1.29 (1.24-1.35) | 19.7 | 1.44 (1.36-1.51) | 20.4 | 1.29 (1.22-1.37) | 8.5 | 1.43 (1.35-1.52) | 8 |
| 10,50,90 | 7 | 10 | 1.26 (1.17-1.35) | 15.5 | 1.29 (1.21-1.39) | 16.3 | 1.33 (1.24-1.44) | 16 | 1.42 (1.32-1.53) | 17 | 1.52 (1.40-1.65) | 17.2 | 1.34 (1.28-1.40) | 17.9 | 1.47 (1.39-1.56) | 18.8 | 1.32 (1.25-1.39) | 16.7 | 1.40 (1.31-1.49) | 8.8 |
| 10,50,90 | 7 | 13 | 1.21 (1.12-1.30) | 15.6 | 1.31 (1.22-1.42) | 15 | 1.34 (1.24-1.46) | 14.6 | 1.43 (1.33-1.54) | 16.4 | 1.57 (1.44-1.72) | 16.2 | 1.33 (1.26-1.40) | 16.5 | 1.48 (1.39-1.58) | 17.5 | 1.30 (1.23-1.38) | 15.6 | 1.37 (1.28-1.46) | 9.3 |
| 10,50,90 | 14 | 7 | 1.25 (1.14-1.36) | 17.2 | 1.22 (1.12-1.34) | 18.2 | 1.14 (1.03-1.27) | 10.1 | 1.12 (1.00-1.25) | 8.8 | 1.49 (1.35-1.64) | 21 | 1.25 (1.18-1.32) | 21.1 | 1.38 (1.29-1.48) | 21.2 | 1.26 (1.17-1.37) | 8.6 | 1.42 (1.31-1.55) | 8.3 |
| 10,50,90 | 14 | 10 | 1.37 (1.25-1.51) | 15.4 | 1.33 (1.19-1.48) | 16.9 | 1.42 (1.28-1.58) | 13.8 | 1.36 (1.22-1.52) | 17.2 | 1.50 (1.34-1.69) | 19.6 | 1.32 (1.24-1.41) | 19.7 | 1.48 (1.36-1.62) | 19.3 | 1.30 (1.22-1.40) | 19.7 | 1.38 (1.26-1.50) | 9.1 |
| 10,50,90 | 14 | 13 | 1.32 (1.18-1.48) | 15.2 | 1.43 (1.27-1.61) | 15.6 | 1.48 (1.30-1.68) | 13 | 1.42 (1.26-1.60) | 15.5 | 1.57 (1.37-1.79) | 18.1 | 1.29 (1.20-1.39) | 19.6 | 1.56 (1.39-1.74) | 10.2 | 1.27 (1.17-1.38) | 17.7 | 1.32 (1.21-1.45) | 13 |
| 10,50,90 | 21 | 7 | 1.23 (1.10-1.37) | 16.3 | 1.09 (0.97-1.22) | 16.5 | 1.04 (0.97-1.11) | 31.2 | 1.17 (1.09-1.25) | 30 | 1.48 (1.31-1.66) | 21.3 | 1.21 (1.13-1.30) | 21.5 | 1.33 (1.23-1.44) | 21.7 | 1.22 (1.10-1.35) | 9.1 | 1.39 (1.25-1.55) | 8.5 |
| 10,50,90 | 21 | 10 | 1.45 (1.28-1.64) | 15.2 | 1.21 (1.04-1.41) | 15.1 | 1.44 (1.24-1.66) | 14.3 | 1.31 (1.12-1.53) | 17.8 | 1.53 (1.31-1.78) | 20.6 | 1.33 (1.21-1.46) | 20.1 | 1.51 (1.34-1.71) | 19.7 | 1.29 (1.18-1.41) | 21.4 | 1.37 (1.21-1.55) | 8.4 |
| 10,50,90 | 21 | 13 | 1.46 (1.24-1.73) | 14.5 | 1.41 (1.17-1.71) | 13.6 | 1.51 (1.25-1.84) | 14.1 | 1.49 (1.24-1.78) | 15.8 | 1.62 (1.33-1.97) | 18.5 | 1.30 (1.17-1.44) | 20.6 | 1.83 (1.53-2.18) | 8.8 | 1.23 (1.09-1.38) | 19.4 | 1.26 (1.12-1.42) | 19.9 |
| 10,75,90 | 7 | 7 | 1.21 (1.14-1.30) | 17.8 | 1.27 (1.18-1.36) | 14.9 | 1.22 (1.15-1.30) | 22.3 | 1.30 (1.19-1.41) | 9.5 | 1.50 (1.37-1.64) | 11.7 | 1.30 (1.25-1.36) | 21.5 | 1.44 (1.37-1.51) | 22.6 | 1.33 (1.25-1.41) | 10.6 | 1.45 (1.37-1.54) | 9 |
| 10,75,90 | 7 | 10 | 1.26 (1.17-1.35) | 16.7 | 1.30 (1.21-1.40) | 16 | 1.35 (1.25-1.45) | 14.8 | 1.43 (1.32-1.54) | 15.7 | 1.53 (1.40-1.67) | 15.6 | 1.35 (1.28-1.41) | 16.7 | 1.48 (1.39-1.58) | 14.1 | 1.33 (1.26-1.40) | 15.6 | 1.41 (1.33-1.49) | 10.8 |
| 10,75,90 | 7 | 13 | 1.21 (1.12-1.30) | 15.7 | 1.32 (1.22-1.42) | 14.9 | 1.35 (1.24-1.47) | 14.1 | 1.45 (1.34-1.57) | 14.2 | 1.57 (1.44-1.72) | 15.7 | 1.35 (1.28-1.43) | 12.6 | 1.50 (1.40-1.61) | 13.1 | 1.31 (1.24-1.39) | 14.7 | 1.39 (1.30-1.49) | 10.1 |
| 10,75,90 | 14 | 7 | 1.25 (1.14-1.36) | 17.6 | 1.23 (1.12-1.36) | 14.3 | 1.18 (1.07-1.31) | 10.9 | 1.13 (1.01-1.27) | 9.4 | 1.52 (1.38-1.68) | 22.8 | 1.30 (1.22-1.38) | 22.9 | 1.41 (1.32-1.51) | 23.6 | 1.33 (1.23-1.44) | 10.3 | 1.45 (1.34-1.57) | 9.5 |
| 10,75,90 | 14 | 10 | 1.36 (1.24-1.50) | 16.6 | 1.33 (1.19-1.48) | 16.9 | 1.44 (1.29-1.60) | 13.7 | 1.36 (1.21-1.52) | 15.6 | 1.48 (1.32-1.66) | 18.5 | 1.33 (1.25-1.43) | 21.1 | 1.51 (1.37-1.66) | 13 | 1.32 (1.23-1.42) | 17 | 1.38 (1.28-1.49) | 13.8 |
| 10,75,90 | 14 | 13 | 1.31 (1.17-1.47) | 15.1 | 1.44 (1.28-1.63) | 14.1 | 1.48 (1.31-1.68) | 13.4 | 1.43 (1.27-1.62) | 14 | 1.56 (1.37-1.79) | 17.5 | 1.32 (1.23-1.42) | 21.9 | 1.58 (1.42-1.77) | 11.7 | 1.28 (1.18-1.40) | 16.4 | 1.34 (1.22-1.48) | 13.3 |
| 10,75,90 | 21 | 7 | 1.22 (1.09-1.36) | 17.3 | 1.10 (0.97-1.24) | 14.4 | 1.05 (0.92-1.20) | 9.9 | 1.14 (1.03-1.26) | 30 | 1.60 (1.41-1.81) | 23.2 | 1.29 (1.19-1.39) | 23.4 | 1.39 (1.28-1.50) | 24.1 | 1.31 (1.18-1.46) | 10.6 | 1.43 (1.28-1.60) | 9.6 |
| 10,75,90 | 21 | 10 | 1.41 (1.24-1.60) | 16.8 | 1.21 (1.04-1.40) | 16.5 | 1.45 (1.25-1.68) | 14.6 | 1.30 (1.11-1.52) | 16.4 | 1.51 (1.29-1.78) | 21.1 | 1.36 (1.24-1.49) | 21.7 | 1.58 (1.38-1.82) | 12 | 1.30 (1.17-1.44) | 15.8 | 1.35 (1.22-1.49) | 15.9 |
| 10,75,90 | 21 | 13 | 1.43 (1.21-1.69) | 15 | 1.43 (1.18-1.74) | 13.2 | 1.54 (1.27-1.85) | 14.8 | 1.47 (1.23-1.76) | 15.7 | 1.63 (1.35-1.98) | 19 | 1.36 (1.22-1.51) | 22.8 | 1.84 (1.54-2.19) | 10.1 | 1.24 (1.09-1.41) | 16.6 | 1.28 (1.12-1.45) | 16.1 |

**Supplementary Figure 1**. Daily time series of total reported deaths and mean air temperature.

**
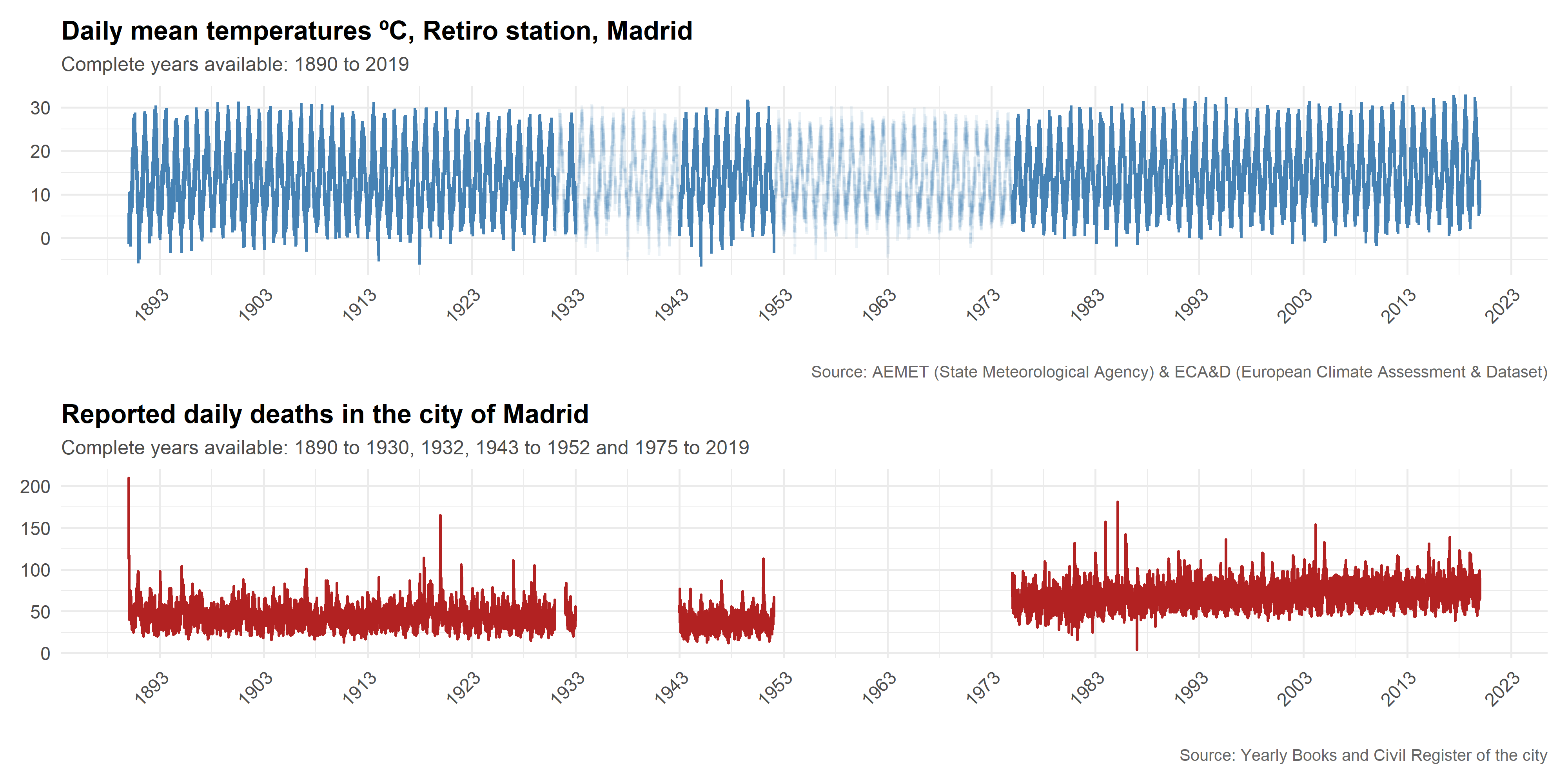
**

**Supplementary Figure 2**. Age- and sex- specific cumulative temperature-mortality associations in Madrid between the 1890s and 2010s.


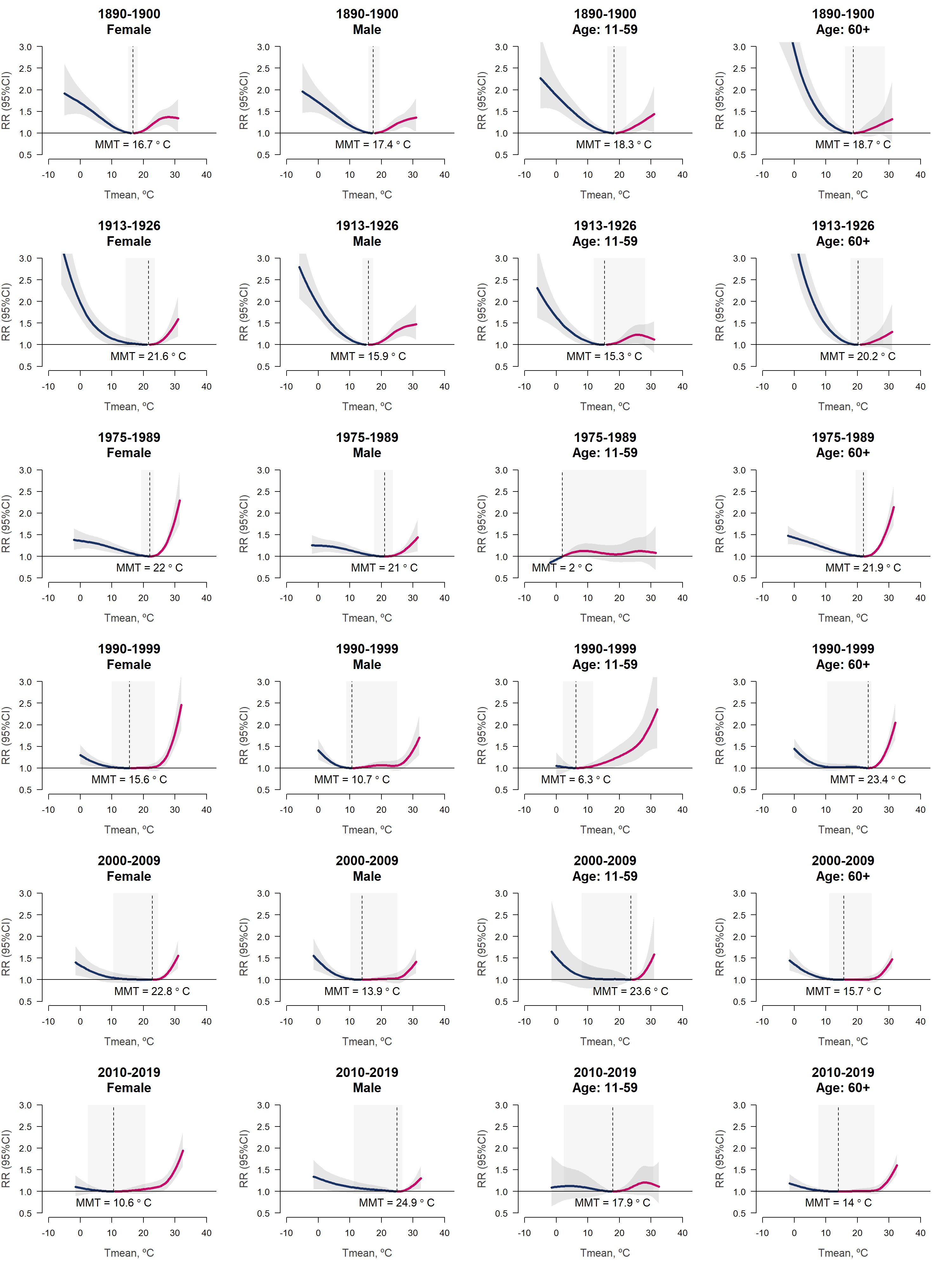


**Supplementary Figure 3**. Evolution of the key climate variables using 30-year moving windows, Retiro station, Madrid.


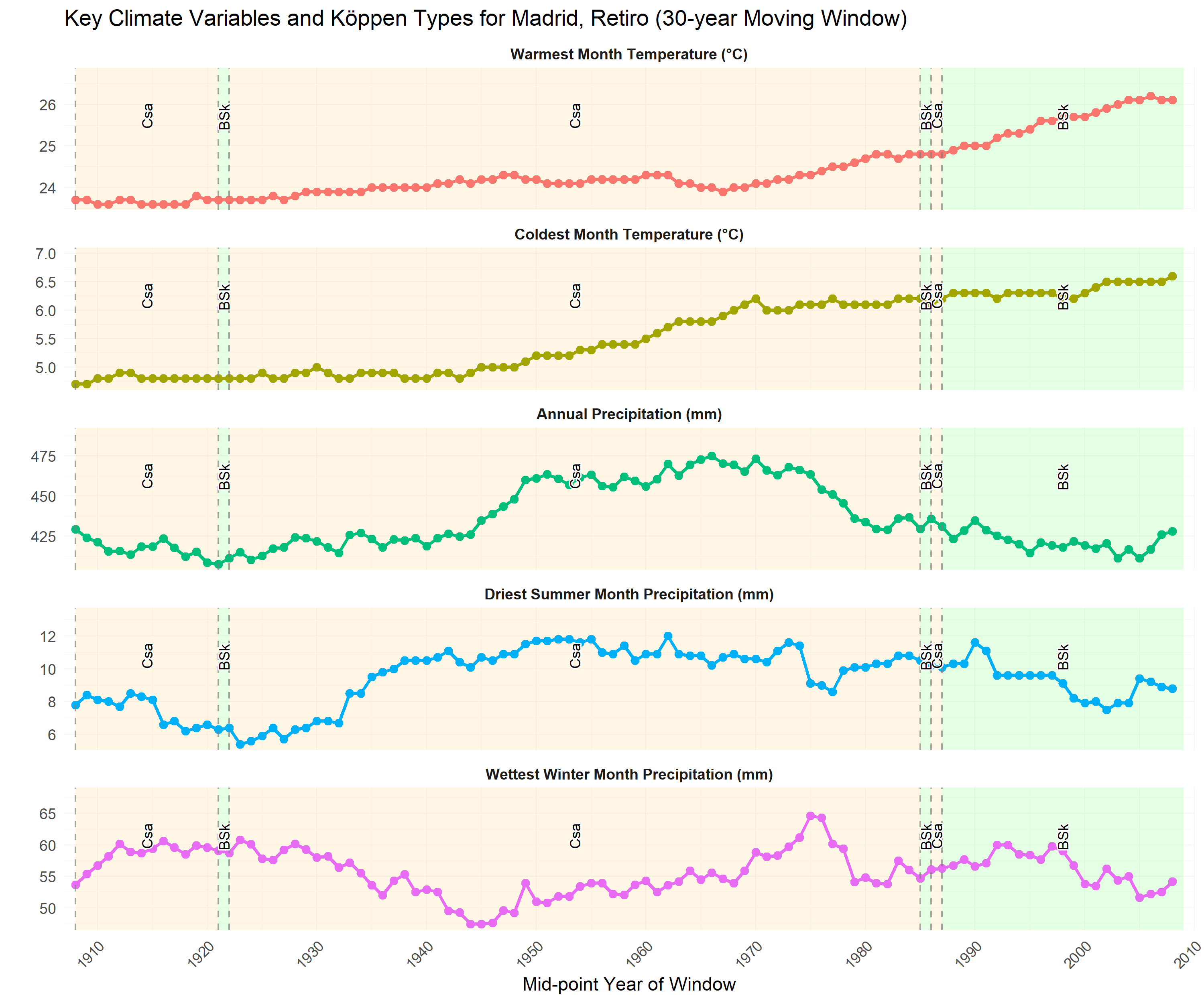


*Annotation on the plot: Köppen–Geiger climate classification, Csa for Hot-summer Mediterranean climate; BSk for Cold semi-arid climate.*

**Supplementary Figure 4**. Lagged relative risks at percentiles P_1_ (cold) and P_99_ (heat) relative to its corresponding MMT in the 1890s and 2010s, by large age groups


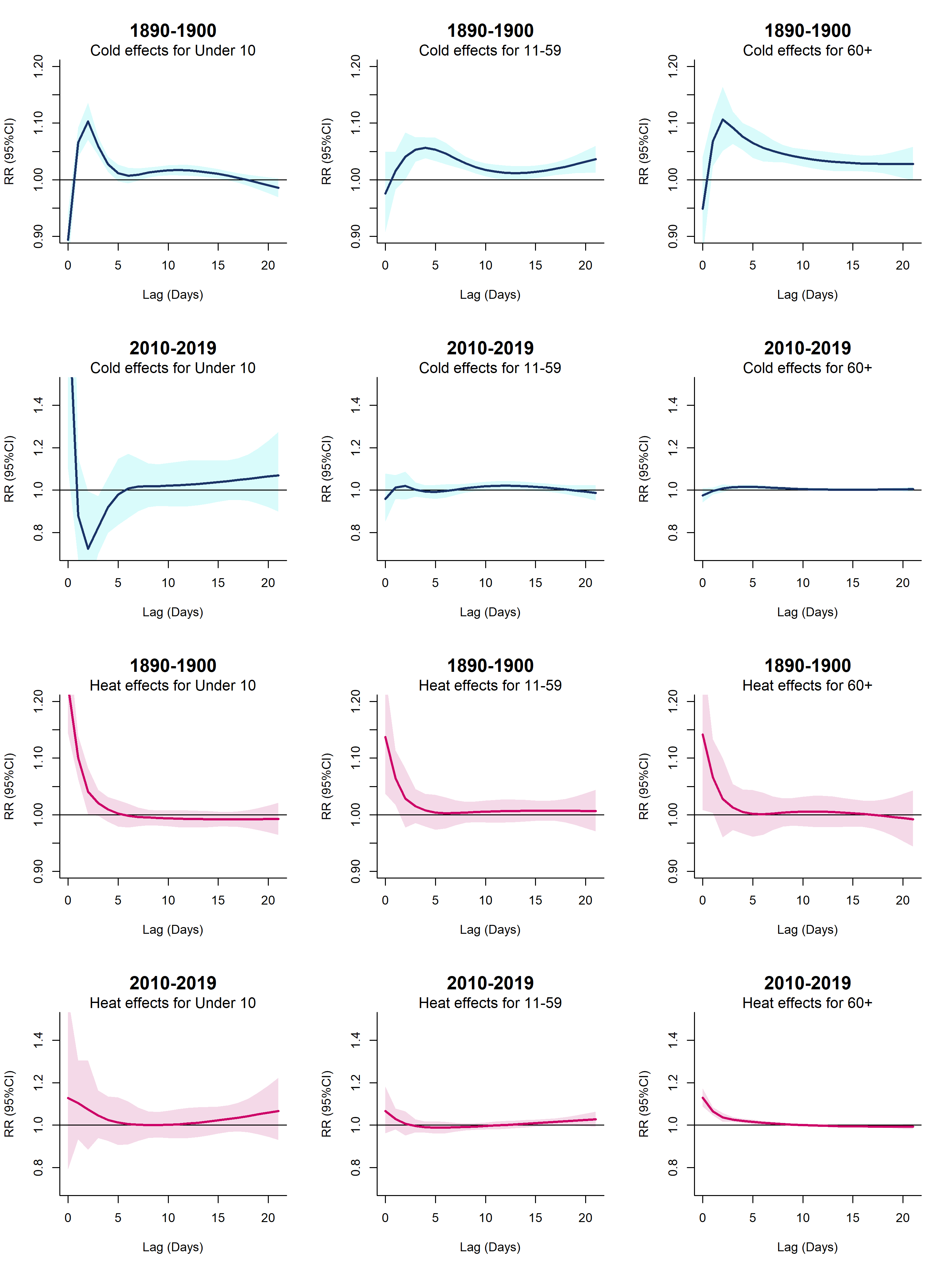


**Supplementary Figure 5**. Geographic location of the astronomical (a,b) and meteorological observatories (c-i) in the Retiro park.


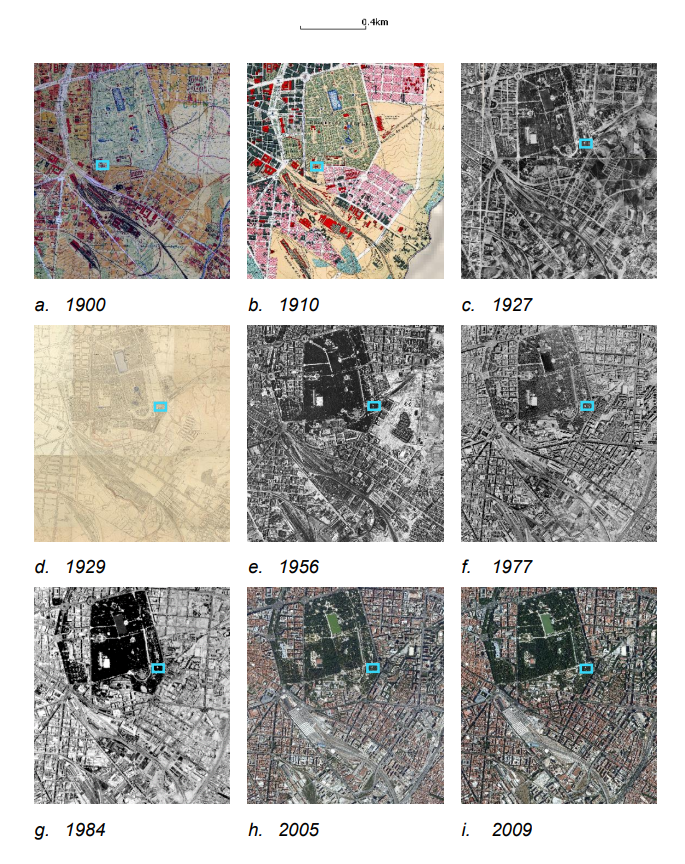

Supplement: Supplementary file 1 — Supplementary Material 1 [file 41598_2026_38595_MOESM1_ESM.docx]
