# Supplementary material for: Exploring 130 years of temperature-related mortality in the city of Madrid
Source: Sci Rep. 2026 Feb 25;16:7641. doi: 10.1038/s41598-026-38595-4 (PMC12936060; doi:10.1038/s41598-026-38595-4)
Supplement: Supplementary file 2 — Supplementary Material 2 [file 41598_2026_38595_MOESM2_ESM.docx]

**Supplementary Table 1.** Attributable mortality fractions by age- and sex- group in Madrid between the 1890s and 2010s.

| **Decade** |  | **Mortality attributable fractions (%)** | | | |
| --- | --- | --- | --- | --- | --- |
|  |  | **Extreme cold** | **Moderate cold** | **Moderate heat** | **Extreme heat** |
| **1890-1900** | 11-59 | 2.57 (1.81- 3.25) | 13.69 (7.54-19.27) | 1.66 (-0.15- 3.40) | 0.81 (0.28- 1.35) |
|  | 60+ | 4.81 (3.69- 5.93) | 20.17 (11.98-27.00) | 1.09 (-0.93- 3.06) | 0.56 (-0.10- 1.20) |
|  | Female | 2.35 (1.76- 3.02) | 11.43 (6.67-15.63) | 3.56 (1.60- 5.36) | 1.18 (0.70- 1.62) |
|  | Male | 2.33 (1.71- 2.93) | 11.85 (7.32-16.01) | 2.45 (0.76- 3.98) | 0.96 (0.50- 1.40) |
| **1913-1926** | 11-59 | 1.80 (1.06- 2.45) | 7.45 (1.77-12.15) | 2.81 (0.12- 4.94) | 0.68 (0.04- 1.23) |
|  | 60+ | 5.71 (4.65- 6.75) | 26.72 (19.14-33.66) | 0.48 (-0.73- 1.55) | 0.42 (-0.09- 0.87) |
|  | Female | 2.53 (1.62- 3.35) | 11.60 (4.11-18.53) | 0.38 (-0.10- 0.84) | 0.64 (0.26- 1.01) |
|  | Male | 2.51 (1.87- 3.13) | 10.82 (6.26-15.01) | 3.64 (1.68- 5.40) | 1.25 (0.71- 1.77) |
| **1975-1989** | 11-59 | 0.21 (-0.01- 0.45) | 0.00 (0.00- 0.00) | 8.00 (-4.46-18.89) | 0.46 (-0.66- 1.42) |
|  | 60+ | 1.51 (1.04- 1.99) | 10.00 (5.82-14.10) | 0.68 (0.33- 1.03) | 1.00 (0.77- 1.24) |
|  | Female | 1.39 (0.81- 1.92) | 10.22 (4.50-15.63) | 0.71 (0.30- 1.11) | 1.09 (0.80- 1.40) |
|  | Male | 1.01 (0.43- 1.52) | 7.23 (2.23-12.03) | 0.48 (-0.24- 1.14) | 0.54 (0.22- 0.87) |
| **1990-1999** | 11-59 | 0.07 (-0.35- 0.46) | 0.01 (-0.14- 0.16) | 11.64 (2.88-19.04) | 2.49 (1.36- 3.54) |
|  | 60+ | 0.92 (0.37- 1.40) | 2.97 (-2.18- 7.93) | 0.64 (0.32- 0.98) | 1.18 (0.83- 1.52) |
|  | Female | 0.71 (0.20- 1.25) | 1.89 (-1.00- 4.62) | 1.43 (-0.76- 3.32) | 1.65 (1.10- 2.19) |
|  | Male | 0.73 (0.33- 1.09) | 0.95 (0.20- 1.76) | 2.52 (-0.76- 5.70) | 0.96 (0.30- 1.51) |
| **2000-2009** | 11-59 | 0.95 (-0.42- 2.13) | 3.30 (-14.22-16.76) | 0.55 (-0.28- 1.45) | 0.81 (0.02- 1.51) |
|  | 60+ | 0.78 (0.36- 1.18) | 2.08 (-0.41- 4.46) | 0.69 (-1.20- 2.57) | 0.73 (0.35- 1.09) |
|  | Female | 0.81 (0.11- 1.44) | 3.18 (-3.55- 9.74) | 0.63 (-0.04- 1.26) | 0.84 (0.46- 1.17) |
|  | Male | 0.85 (0.39- 1.30) | 1.92 (-0.28- 4.10) | 1.10 (-1.88- 3.69) | 0.66 (0.13- 1.16) |
| **2010-2019** | 11-59 | 0.50 (-0.87- 1.77) | 3.55 (-6.73-12.70) | 2.88 (-1.69- 6.98) | 0.64 (-0.44- 1.67) |
|  | 60+ | 0.32 (-0.04- 0.67) | 0.76 (-1.10- 2.55) | 0.89 (-1.41- 3.07) | 0.87 (0.49- 1.22) |
|  | Female | 0.16 (-0.22- 0.53) | 0.22 (-0.73- 1.07) | 3.44 (-0.73- 7.72) | 1.42 (0.89- 1.90) |
|  | Male | 0.83 (0.06- 1.49) | 5.74 (-2.12-12.70) | 0.23 (-0.11- 0.56) | 0.44 (0.14- 0.74) |

Moderate cold (P_5_ vs. MMT); Extreme cold (P_1_ vs. P_5_); Moderate heat (P_95_ vs MMT); Extreme heat (P_99_ vs. P_95_).
