# Supplementary figures and images for: Exploring 130 years of temperature-related mortality in the city of Madrid
Source: Sci Rep. 2026 Feb 25;16:7641. doi: 10.1038/s41598-026-38595-4 (PMC12936060; doi:10.1038/s41598-026-38595-4)

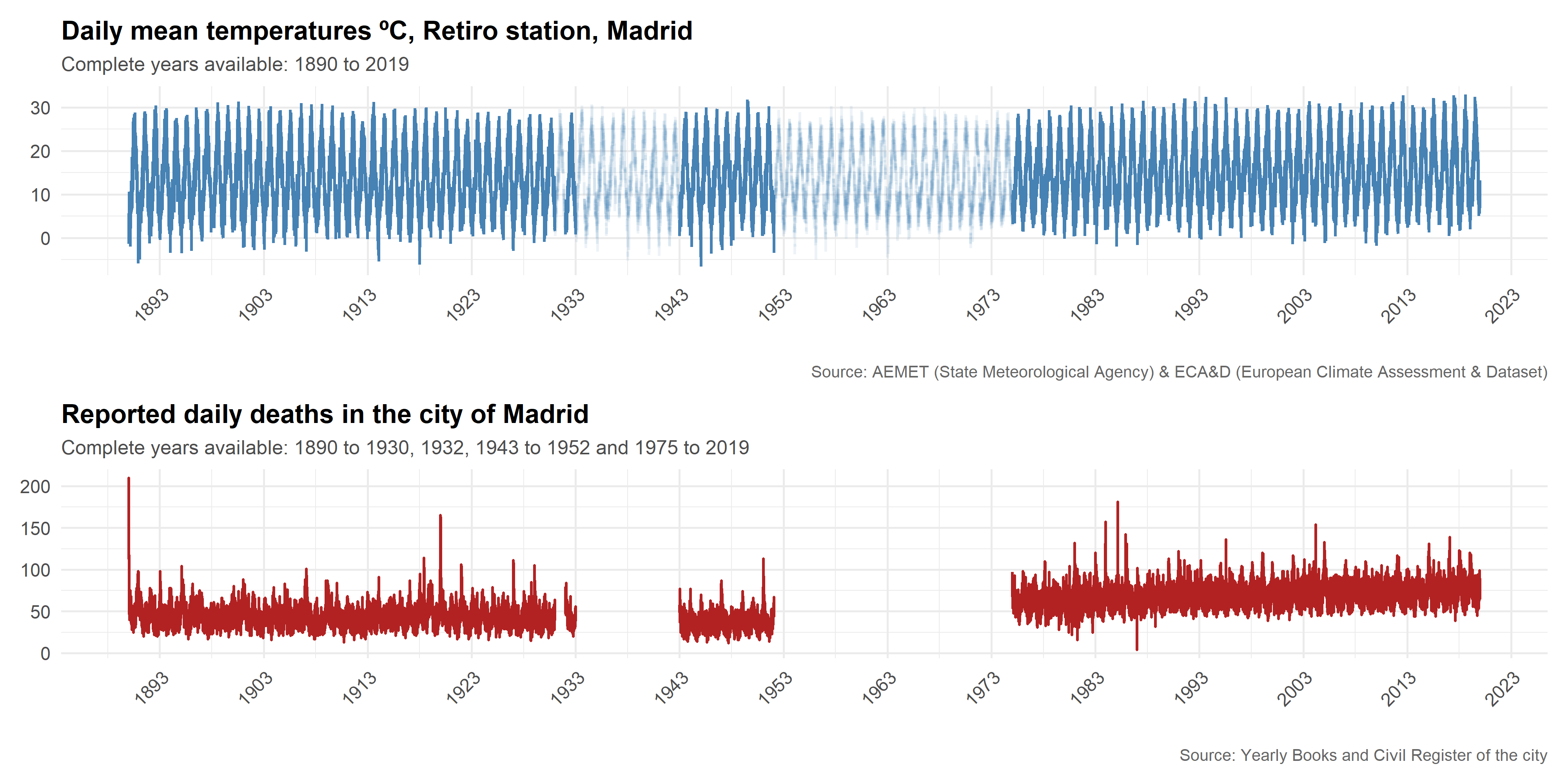

Supplement: Supplementary file 3 — Supplementary Material 3 [file 41598_2026_38595_MOESM3_ESM.png]

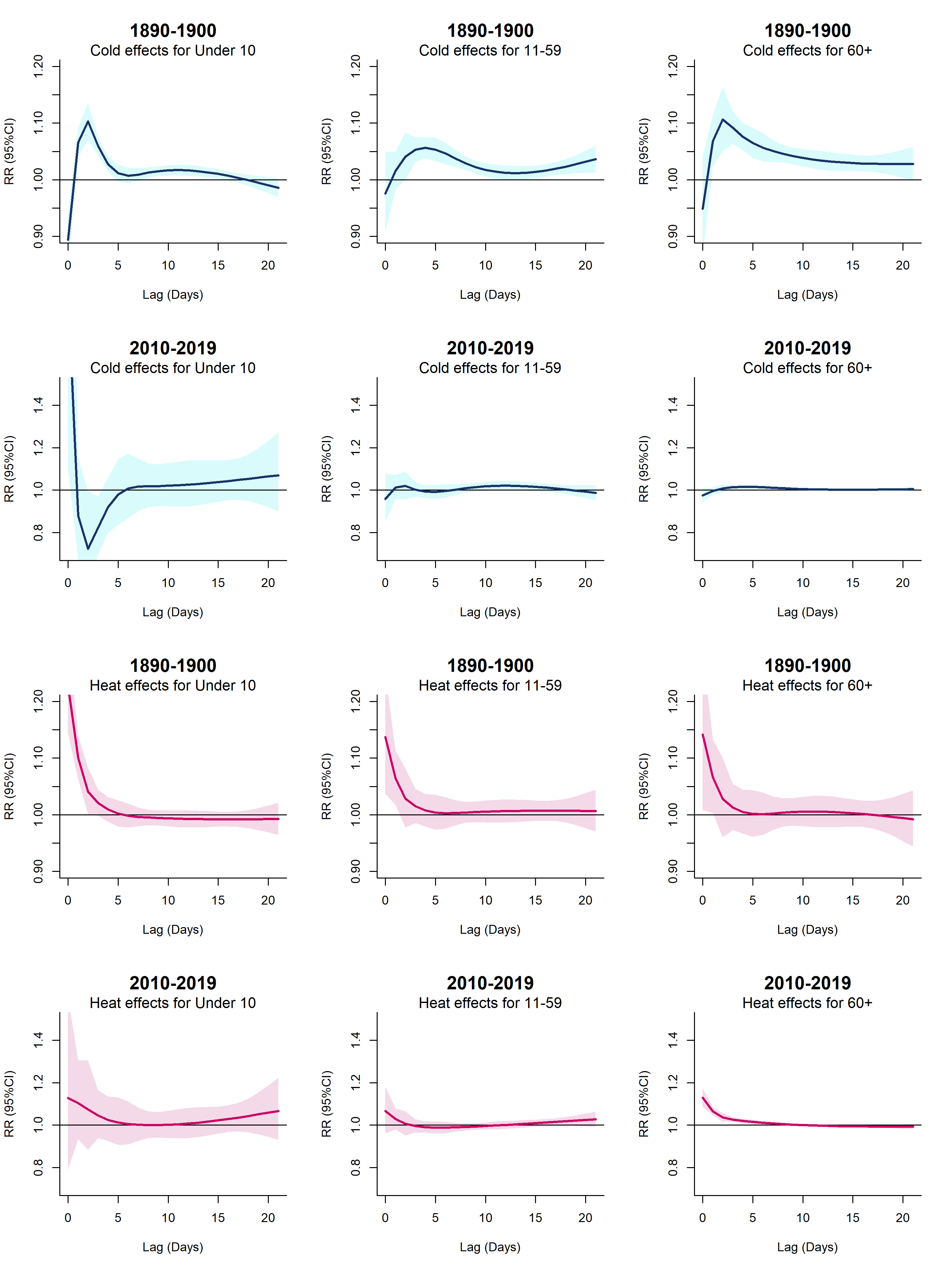

Supplement: Supplementary file 4 — Supplementary Material 4 [file 41598_2026_38595_MOESM4_ESM.png]

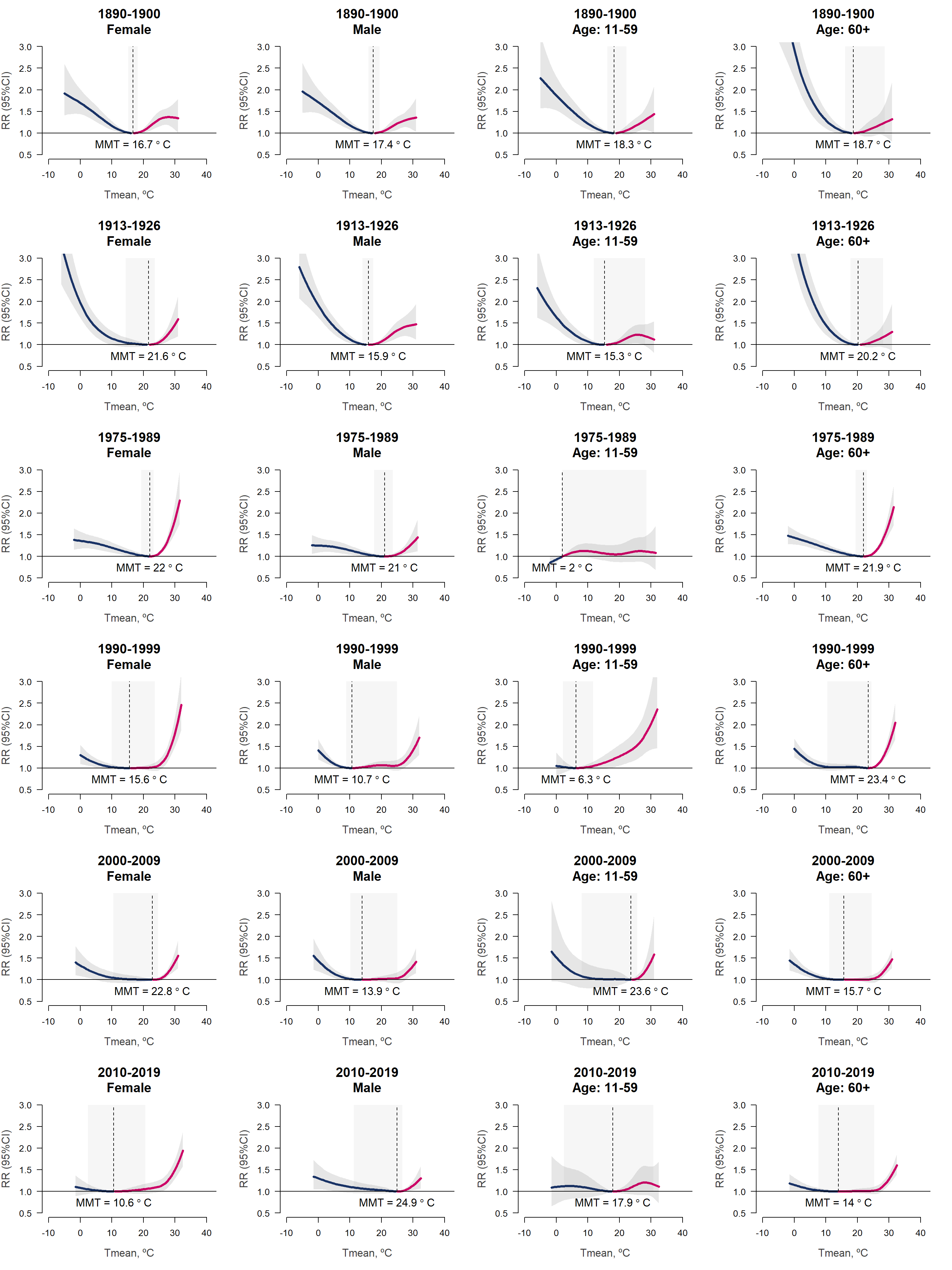

Supplement: Supplementary file 5 — Supplementary Material 5 [file 41598_2026_38595_MOESM5_ESM.png]

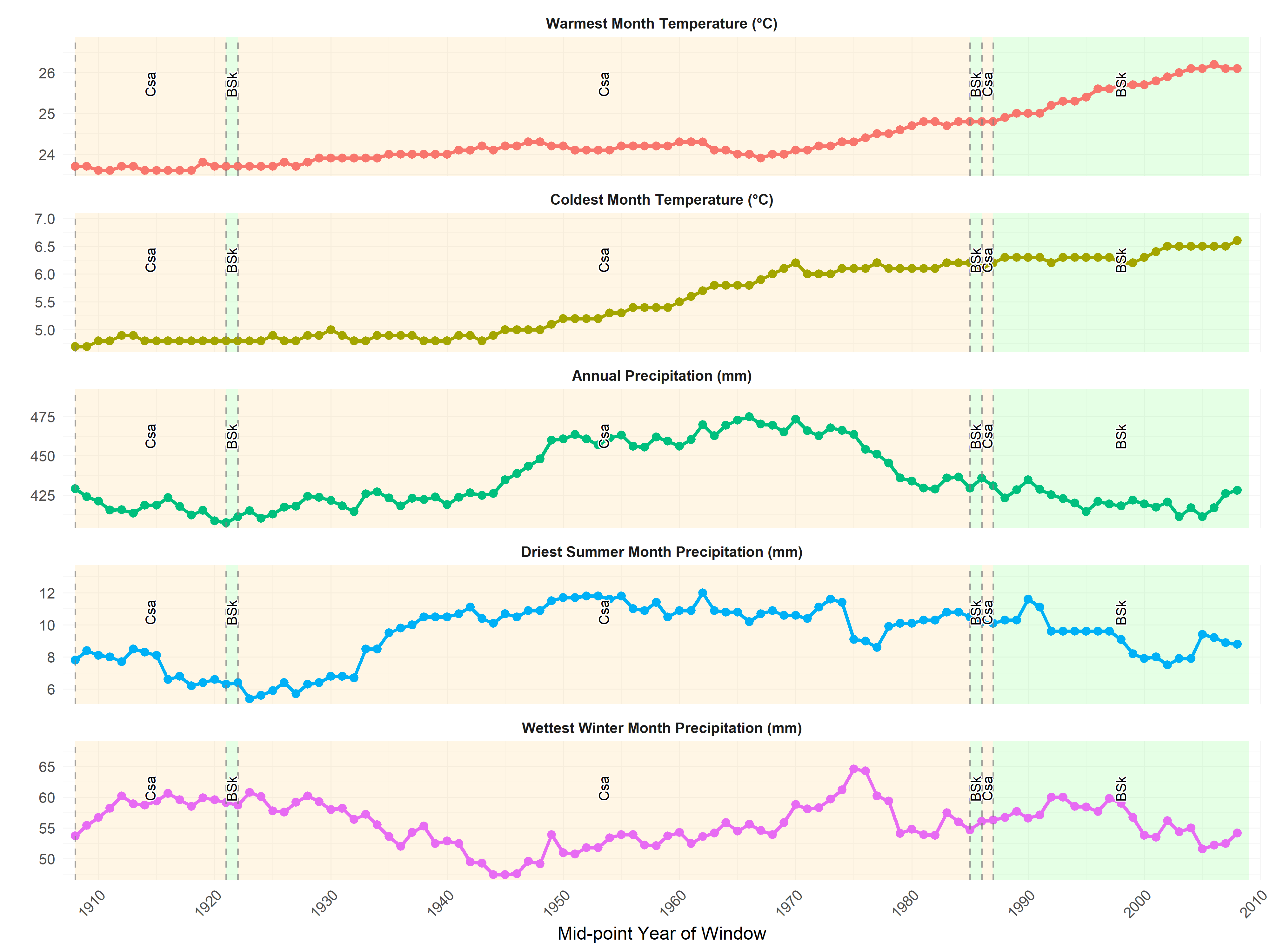

Supplement: Supplementary file 6 — Supplementary Material 6 [file 41598_2026_38595_MOESM6_ESM.png]

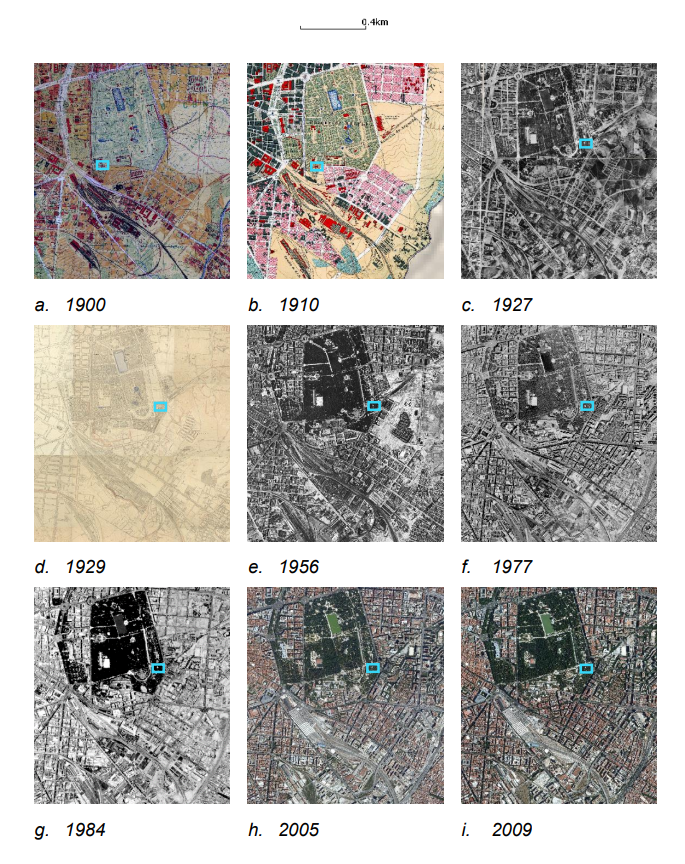

Supplement: Supplementary file 8 — Supplementary Material 8 [file 41598_2026_38595_MOESM8_ESM.png]
