# Supplementary material for: Exploring 130 years of temperature-related mortality in the city of Madrid
Source: Sci Rep. 2026 Feb 25;16:7641. doi: 10.1038/s41598-026-38595-4 (PMC12936060; doi:10.1038/s41598-026-38595-4)
Supplement: Supplementary file 7 — Supplementary Material 7 [file 41598_2026_38595_MOESM7_ESM.docx]

**Supplementary Table 2.** Sensitivity analysis on knots for temperature distribution, lag duration, and number of degrees of freedom (df).

|  |  |  | **1890-1899** | | **1900-1909** | | **1910-1919** | | **1920-1929** | | **1943-1951** | | **1975-1989** | | **1990-1999** | | **2000-2009** | | **2010-2019** | |
| --- | --- | --- | --- | --- | --- | --- | --- | --- | --- | --- | --- | --- | --- | --- | --- | --- | --- | --- | --- | --- |
| Knots | Lag | df | RR at 28.5ºC | MMT | RR at 29.0ºC | MMT | RR at 28.4ºC | MMT | RR at 27.4ºC | MMT | RR at 28.4ºC | MMT | RR at 28.6ºC | MMT | RR at 30.1ºC | MMT | RR at 29.5ºC | MMT | RR at 30.8ºC | MMT |
| 10,25,75,90 | 7 | 7 | 1.21 (1.13-1.30) | 16.4 | 1.27 (1.18-1.36) | 14.4 | 1.22 (1.15-1.30) | 19.5 | 1.30 (1.20-1.42) | 9.5 | 1.50 (1.39-1.62) | 19.5 | 1.31 (1.25-1.37) | 21.4 | 1.44 (1.37-1.51) | 22.4 | 1.33 (1.25-1.41) | 10.6 | 1.45 (1.37-1.54) | 9.5 |
| 10,25,75,90 | 7 | 10 | 1.26 (1.17-1.36) | 14.6 | 1.30 (1.21-1.40) | 15.6 | 1.34 (1.25-1.45) | 15.3 | 1.43 (1.32-1.54) | 15 | 1.53 (1.41-1.66) | 17.1 | 1.35 (1.28-1.41) | 16.5 | 1.48 (1.39-1.59) | 13.6 | 1.34 (1.26-1.42) | 14.3 | 1.41 (1.32-1.50) | 8.2 |
| 10,25,75,90 | 7 | 13 | 1.21 (1.12-1.31) | 14.8 | 1.32 (1.22-1.42) | 14.7 | 1.35 (1.24-1.47) | 13.9 | 1.49 (1.37-1.63) | 11.5 | 1.58 (1.44-1.72) | 16.2 | 1.36 (1.28-1.45) | 11.9 | 1.51 (1.40-1.62) | 12.8 | 1.33 (1.25-1.42) | 13 | 1.39 (1.30-1.49) | 10.5 |
| 10,25,75,90 | 14 | 7 | 1.24 (1.14-1.36) | 16.8 | 1.25 (1.13-1.38) | 12.8 | 1.16 (1.04-1.28) | 12 | 1.11 (0.99-1.25) | 9 | 1.49 (1.35-1.65) | 21.9 | 1.32 (1.24-1.40) | 23.2 | 1.41 (1.32-1.51) | 23.6 | 1.32 (1.21-1.43) | 9.8 | 1.45 (1.34-1.58) | 9.9 |
| 10,25,75,90 | 14 | 10 | 1.37 (1.24-1.51) | 15.1 | 1.33 (1.19-1.49) | 15.9 | 1.43 (1.28-1.58) | 14.7 | 1.35 (1.21-1.51) | 17 | 1.49 (1.33-1.68) | 18.9 | 1.34 (1.25-1.44) | 22 | 1.51 (1.37-1.67) | 12.8 | 1.32 (1.23-1.42) | 18.2 | 1.37 (1.28-1.47) | 17 |
| 10,25,75,90 | 14 | 13 | 1.32 (1.17-1.48) | 14.9 | 1.46 (1.28-1.66) | 13.3 | 1.48 (1.30-1.68) | 13.5 | 1.44 (1.26-1.64) | 13.4 | 1.57 (1.38-1.80) | 18.1 | 1.33 (1.24-1.44) | 22.8 | 1.59 (1.42-1.78) | 11.7 | 1.30 (1.18-1.43) | 13.6 | 1.34 (1.22-1.48) | 13.7 |
| 10,25,75,90 | 21 | 7 | 1.22 (1.09-1.36) | 16.4 | 1.13 (0.99-1.29) | 12.6 | 1.06 (0.92-1.22) | 10.1 | 1.17 (1.06-1.30) | 30 | 1.55 (1.36-1.76) | 22.8 | 1.33 (1.23-1.44) | 23.7 | 1.39 (1.28-1.50) | 24.1 | 1.28 (1.14-1.42) | 9.1 | 1.43 (1.28-1.60) | 9.8 |
| 10,25,75,90 | 21 | 10 | 1.41 (1.24-1.60) | 15.9 | 1.21 (1.04-1.41) | 15.2 | 1.44 (1.25-1.66) | 15.8 | 1.30 (1.12-1.52) | 17.7 | 1.50 (1.28-1.76) | 19.8 | 1.38 (1.26-1.52) | 22.9 | 1.60 (1.38-1.84) | 11.8 | 1.30 (1.18-1.43) | 20.5 | 1.36 (1.24-1.49) | 20.7 |
| 10,25,75,90 | 21 | 13 | 1.42 (1.20-1.68) | 15.5 | 1.48 (1.21-1.81) | 11.8 | 1.53 (1.27-1.85) | 15.1 | 1.46 (1.22-1.74) | 16.1 | 1.64 (1.35-1.99) | 18.9 | 1.39 (1.25-1.55) | 23.6 | 1.85 (1.55-2.20) | 10.3 | 1.23 (1.10-1.39) | 18.5 | 1.28 (1.13-1.44) | 19.3 |
| 10,50,90 | 7 | 7 | 1.21 (1.13-1.29) | 17.3 | 1.26 (1.18-1.35) | 17.1 | 1.23 (1.15-1.31) | 19.4 | 1.27 (1.17-1.38) | 8.5 | 1.50 (1.39-1.62) | 19.7 | 1.29 (1.24-1.35) | 19.7 | 1.44 (1.36-1.51) | 20.4 | 1.29 (1.22-1.37) | 8.5 | 1.43 (1.35-1.52) | 8 |
| 10,50,90 | 7 | 10 | 1.26 (1.17-1.35) | 15.5 | 1.29 (1.21-1.39) | 16.3 | 1.33 (1.24-1.44) | 16 | 1.42 (1.32-1.53) | 17 | 1.52 (1.40-1.65) | 17.2 | 1.34 (1.28-1.40) | 17.9 | 1.47 (1.39-1.56) | 18.8 | 1.32 (1.25-1.39) | 16.7 | 1.40 (1.31-1.49) | 8.8 |
| 10,50,90 | 7 | 13 | 1.21 (1.12-1.30) | 15.6 | 1.31 (1.22-1.42) | 15 | 1.34 (1.24-1.46) | 14.6 | 1.43 (1.33-1.54) | 16.4 | 1.57 (1.44-1.72) | 16.2 | 1.33 (1.26-1.40) | 16.5 | 1.48 (1.39-1.58) | 17.5 | 1.30 (1.23-1.38) | 15.6 | 1.37 (1.28-1.46) | 9.3 |
| 10,50,90 | 14 | 7 | 1.25 (1.14-1.36) | 17.2 | 1.22 (1.12-1.34) | 18.2 | 1.14 (1.03-1.27) | 10.1 | 1.12 (1.00-1.25) | 8.8 | 1.49 (1.35-1.64) | 21 | 1.25 (1.18-1.32) | 21.1 | 1.38 (1.29-1.48) | 21.2 | 1.26 (1.17-1.37) | 8.6 | 1.42 (1.31-1.55) | 8.3 |
| 10,50,90 | 14 | 10 | 1.37 (1.25-1.51) | 15.4 | 1.33 (1.19-1.48) | 16.9 | 1.42 (1.28-1.58) | 13.8 | 1.36 (1.22-1.52) | 17.2 | 1.50 (1.34-1.69) | 19.6 | 1.32 (1.24-1.41) | 19.7 | 1.48 (1.36-1.62) | 19.3 | 1.30 (1.22-1.40) | 19.7 | 1.38 (1.26-1.50) | 9.1 |
| 10,50,90 | 14 | 13 | 1.32 (1.18-1.48) | 15.2 | 1.43 (1.27-1.61) | 15.6 | 1.48 (1.30-1.68) | 13 | 1.42 (1.26-1.60) | 15.5 | 1.57 (1.37-1.79) | 18.1 | 1.29 (1.20-1.39) | 19.6 | 1.56 (1.39-1.74) | 10.2 | 1.27 (1.17-1.38) | 17.7 | 1.32 (1.21-1.45) | 13 |
| 10,50,90 | 21 | 7 | 1.23 (1.10-1.37) | 16.3 | 1.09 (0.97-1.22) | 16.5 | 1.04 (0.97-1.11) | 31.2 | 1.17 (1.09-1.25) | 30 | 1.48 (1.31-1.66) | 21.3 | 1.21 (1.13-1.30) | 21.5 | 1.33 (1.23-1.44) | 21.7 | 1.22 (1.10-1.35) | 9.1 | 1.39 (1.25-1.55) | 8.5 |
| 10,50,90 | 21 | 10 | 1.45 (1.28-1.64) | 15.2 | 1.21 (1.04-1.41) | 15.1 | 1.44 (1.24-1.66) | 14.3 | 1.31 (1.12-1.53) | 17.8 | 1.53 (1.31-1.78) | 20.6 | 1.33 (1.21-1.46) | 20.1 | 1.51 (1.34-1.71) | 19.7 | 1.29 (1.18-1.41) | 21.4 | 1.37 (1.21-1.55) | 8.4 |
| 10,50,90 | 21 | 13 | 1.46 (1.24-1.73) | 14.5 | 1.41 (1.17-1.71) | 13.6 | 1.51 (1.25-1.84) | 14.1 | 1.49 (1.24-1.78) | 15.8 | 1.62 (1.33-1.97) | 18.5 | 1.30 (1.17-1.44) | 20.6 | 1.83 (1.53-2.18) | 8.8 | 1.23 (1.09-1.38) | 19.4 | 1.26 (1.12-1.42) | 19.9 |
| 10,75,90 | 7 | 7 | 1.21 (1.14-1.30) | 17.8 | 1.27 (1.18-1.36) | 14.9 | 1.22 (1.15-1.30) | 22.3 | 1.30 (1.19-1.41) | 9.5 | 1.50 (1.37-1.64) | 11.7 | 1.30 (1.25-1.36) | 21.5 | 1.44 (1.37-1.51) | 22.6 | 1.33 (1.25-1.41) | 10.6 | 1.45 (1.37-1.54) | 9 |
| 10,75,90 | 7 | 10 | 1.26 (1.17-1.35) | 16.7 | 1.30 (1.21-1.40) | 16 | 1.35 (1.25-1.45) | 14.8 | 1.43 (1.32-1.54) | 15.7 | 1.53 (1.40-1.67) | 15.6 | 1.35 (1.28-1.41) | 16.7 | 1.48 (1.39-1.58) | 14.1 | 1.33 (1.26-1.40) | 15.6 | 1.41 (1.33-1.49) | 10.8 |
| 10,75,90 | 7 | 13 | 1.21 (1.12-1.30) | 15.7 | 1.32 (1.22-1.42) | 14.9 | 1.35 (1.24-1.47) | 14.1 | 1.45 (1.34-1.57) | 14.2 | 1.57 (1.44-1.72) | 15.7 | 1.35 (1.28-1.43) | 12.6 | 1.50 (1.40-1.61) | 13.1 | 1.31 (1.24-1.39) | 14.7 | 1.39 (1.30-1.49) | 10.1 |
| 10,75,90 | 14 | 7 | 1.25 (1.14-1.36) | 17.6 | 1.23 (1.12-1.36) | 14.3 | 1.18 (1.07-1.31) | 10.9 | 1.13 (1.01-1.27) | 9.4 | 1.52 (1.38-1.68) | 22.8 | 1.30 (1.22-1.38) | 22.9 | 1.41 (1.32-1.51) | 23.6 | 1.33 (1.23-1.44) | 10.3 | 1.45 (1.34-1.57) | 9.5 |
| 10,75,90 | 14 | 10 | 1.36 (1.24-1.50) | 16.6 | 1.33 (1.19-1.48) | 16.9 | 1.44 (1.29-1.60) | 13.7 | 1.36 (1.21-1.52) | 15.6 | 1.48 (1.32-1.66) | 18.5 | 1.33 (1.25-1.43) | 21.1 | 1.51 (1.37-1.66) | 13 | 1.32 (1.23-1.42) | 17 | 1.38 (1.28-1.49) | 13.8 |
| 10,75,90 | 14 | 13 | 1.31 (1.17-1.47) | 15.1 | 1.44 (1.28-1.63) | 14.1 | 1.48 (1.31-1.68) | 13.4 | 1.43 (1.27-1.62) | 14 | 1.56 (1.37-1.79) | 17.5 | 1.32 (1.23-1.42) | 21.9 | 1.58 (1.42-1.77) | 11.7 | 1.28 (1.18-1.40) | 16.4 | 1.34 (1.22-1.48) | 13.3 |
| 10,75,90 | 21 | 7 | 1.22 (1.09-1.36) | 17.3 | 1.10 (0.97-1.24) | 14.4 | 1.05 (0.92-1.20) | 9.9 | 1.14 (1.03-1.26) | 30 | 1.60 (1.41-1.81) | 23.2 | 1.29 (1.19-1.39) | 23.4 | 1.39 (1.28-1.50) | 24.1 | 1.31 (1.18-1.46) | 10.6 | 1.43 (1.28-1.60) | 9.6 |
| 10,75,90 | 21 | 10 | 1.41 (1.24-1.60) | 16.8 | 1.21 (1.04-1.40) | 16.5 | 1.45 (1.25-1.68) | 14.6 | 1.30 (1.11-1.52) | 16.4 | 1.51 (1.29-1.78) | 21.1 | 1.36 (1.24-1.49) | 21.7 | 1.58 (1.38-1.82) | 12 | 1.30 (1.17-1.44) | 15.8 | 1.35 (1.22-1.49) | 15.9 |
| 10,75,90 | 21 | 13 | 1.43 (1.21-1.69) | 15 | 1.43 (1.18-1.74) | 13.2 | 1.54 (1.27-1.85) | 14.8 | 1.47 (1.23-1.76) | 15.7 | 1.63 (1.35-1.98) | 19 | 1.36 (1.22-1.51) | 22.8 | 1.84 (1.54-2.19) | 10.1 | 1.24 (1.09-1.41) | 16.6 | 1.28 (1.12-1.45) | 16.1 |
