# Supplementary material for: Exploring 130 years of temperature-related mortality in the city of Madrid
Source: Sci Rep. 2026 Feb 25;16:7641. doi: 10.1038/s41598-026-38595-4 (PMC12936060; doi:10.1038/s41598-026-38595-4)
Supplement: Supplementary file 9 — Supplementary Material 9 [file 41598_2026_38595_MOESM9_ESM.docx]

Please find the titles of the supplementary items below. The same titles are provided in the file **dordanovich_130years-madrid-SI.docx** next to the corresponding tables/figures.

**Supplementary Table 1.** Attributable mortality fractions by age- and sex- group in Madrid between the 1890s and 2010s

**Supplementary Table 2.** Sensitivity analysis on knots for temperature distribution, lag duration, and number of degrees of freedom (df).

**Supplementary Figure 1**. Daily time series of total reported deaths and mean air temperature.

**Supplementary Figure 2**. Age- and sex- specific cumulative temperature-mortality associations in Madrid between the 1890s and 2010s.

**Supplementary Figure 3**. Evolution of the key climate variables using 30-year moving windows, Retiro station, Madrid.

**Supplementary Figure 4**. Lagged relative risks at percentiles P_1_ (cold) and P_99_ (heat) relative to its corresponding MMT in the 1890s and 2010s, by large age groups

**Supplementary Figure 5**. Geographic location of the astronomical (a,b) and meteorological observatories (c-i) in the Retiro park.
